# Chiral lanthanide lumino-glass for a circularly polarized light security device

**DOI:** 10.1038/s42004-020-00366-1

**Published:** 2020-08-25

**Authors:** Yuichi Kitagawa, Satoshi Wada, M. D. Jahidul Islam, Kenichiro Saita, Masayuki Gon, Koji Fushimi, Kazuo Tanaka, Satoshi Maeda, Yasuchika Hasegawa

**Affiliations:** 1 Faculty of Engineering, Hokkaido University, N13 W8, Kita-ku, Sapporo, Hokkaido 060–8628 Japan; 2 Institute for Chemical Reaction Design and Discovery (WPI-ICReDD), Hokkaido University, N21 W10, Sapporo, Hokkaido 001-0021 Japan; 3 Faculty of Science, Department of Chemistry, Hokkaido University, Sapporo, 060-0810 Japan; 4 Graduate School of Engineering, Kyoto University, Katsura, Nishikyo-ku, Kyoto, 615-8510 Japan

**Keywords:** Sensors and biosensors, Inorganic chemistry, Nonlinear optics, Glasses, Optical materials

## Abstract

Artificial light plays an essential role in information technologies such as optical telecommunications, data storage, security features, and the display of information. Here, we show a chiral lanthanide lumino-glass with extra-large circularly polarized luminescence (CPL) for advanced photonic security device applications. The chiral lanthanide glass is composed of a europium complex with the chiral (+)-3-(trifluoroacetyl)camphor ligand and the achiral glass promoter tris(2,6-dimethoxyphenyl)phosphine oxide ligand. The glass phase transition behavior of the Eu(III) complex is characterized using differential scanning calorimetry. The transparent amorphous glass shows CPL with extra-large dissymmetry factor of *g*_CPL_ = 1.2. The brightness of the lumino-glass is one thousand times larger than that of Eu(III) luminophores embedded in polymer films of the same thickness at a Eu(III) concentration of 1 mM. The application of the chiral lanthanide lumino-glass in an advanced security paint is demonstrated.

## Abstract (web summary)

Circularly polarized luminescence from chiral organic molecules is used in devices such as security tags, lasers, or data storage. Here, the authors use (+)-3-(trifluoroacetyl)camphor and a glass-promoting phosphine oxide ligand to achieve a chiral Eu(III) lumino-glass.

## Introduction

Artificial light plays an essential role in information technologies such as optical telecommunications, data storage, security features, and the display of information^1^. Organic-based luminophores that allow precise control of information have been extensively studied for the development of light-based information technologies^2,3^. This precise control originates from the molecular quantum design of organic luminophores^4–6^. The combination of the molecules with quantum design and flexible amorphous materials is expected to result in advancements in photo-science and -technology for industrial applications such as flexible organic light-emitting diodes (OLED)^7–9^.

In this work, we focus on an optical information technology based on chiral luminescent molecules. Recently, circularly polarized luminescence (CPL) from chiral organic molecules has attracted considerable attention because of its applications in sophisticated photo-information technologies, such as security tags, lasers, data storage, and organic electroluminescent (EL) devices for three-dimensional displays^10–16^. The CPL phenomena are characterized by the differential emission of right- and left-handed circularly polarized light. The magnitude of CPL is given by the following equation^17,18^:

1 $${{g}}_{{\mathrm{CPL}}} = 2\frac{{I_{\mathrm{L}} - I_{\mathrm{R}}}}{{I_{\mathrm{L}} + I_{\mathrm{R}}}}$$

where *I*_L_ and *I*_R_ are the emission intensities of the left- and right-handed CPL, respectively.

In particular, lanthanide complexes containing chiral organic ligands exhibit dissymmetry factors (*g*_CPL_) approximately a thousand times larger than those of chiral organic molecules^10–13,19–22^. Parker prepared anion sensors using the CPL signals of mononuclear Eu(III) and Tb(III) complexes with chiral ligands incorporating an azaxanthone sensitizer^23^. Muller and colleagues^24^ observed an exceptionally large CPL in an Eu(III)-Cs(I) system with characteristic chiral β-diketonate ligands containing a camphor framework (tetrakis(3-heptafluorobutylryl-(+)-camphorato) (*g*_CPL_ = −1.38)^24^, although the emission quantum yield was extremely low (*Φ*_tot_ < 1.0%)^25^. Recently, we found that phosphine oxide ligands improve the quantum emission yield by controlling the energy quenching state for a chiral Eu(III) complex with camphor (*Φ*_tot_ > 10%)^26^. The use of well-designed phosphine oxide ligands is a key strategy for the construction of chiral Eu(III) complexes with large *g*_CPL_ and *Φ*_tot_ values for advanced CPL luminophore applications.

**Fig. 1:**
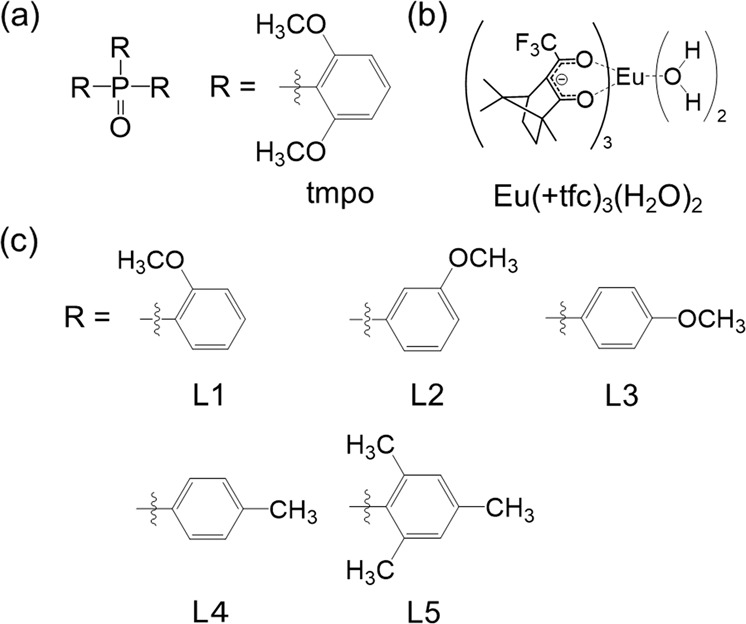
Molecular structures. Chemical structures of tmpo ligand (**a**), Eu(+tfc)_3_(H_2_O)_2_ (**b**), L1, L2, L3, L4, and L5 ligands (**c**).

Herein, we prepare a bulky phosphine oxide ligand with six methoxy groups, tmpo (tris(2,6-dimethoxyphenyl)phosphine oxide, Fig. 1a), for the construction of complexes with large g_CPL_ and *Φ*_tot_ values. The bulky ligand coordinates weakly to the Eu(III) ion; such weak coordination has been reported to be related to the enhancement of CPL^22^. The tmpo ligand is also expected to promote glass formation due to the multiple coordination sites provided by its phosphine oxide (P=O)^26^ and methoxy groups (O–Me)^27^, which is beneficial for the fabrication of transparent materials. The ligand (±)-3-(trifluoroacetyl)camphor (±tfc) is selected to achieve a chiral ligand arrangement around the Eu center (Fig. 1b). The chiral Eu(III) complexes with phosphine oxide ligands are prepared by casting Eu(±tfc)_3_(H_2_O)_2_ and tmpo ligands on a substrate. The glass phase transition behavior of the Eu(III) complex (Eu(±tfc)-tmpo) is characterized using differential scanning calorimetry. The resulting chiral Eu(III) lumino-glass exhibits extraordinary CPL intensity (*g*_CPL_ = 1.2) and a relatively high emission quantum yield (*Φ*_tot_ = 13 %). Chiral Eu(III) complexes with other phosphine oxide ligands (Fig. 1c, L1: Tris(2-methoxyphenyl)phosphine oxide, L2: Tris(3-methoxyphenyl)phosphine oxide, L3: tris(4-methoxyphenyl)phosphine oxide, L4: tris(4-methylphenyl)phosphine oxide, L5: tris(2,4,6-trimethylphenyl)phosphine oxide, Supplementary Methods) are also prepared for comparison of their photophysical properties. Based on spectroscopic analyses, we determine that the extra-large *g*_CPL_ of Eu(±tfc)-tmpo originates from its characteristic symmetric coordination structure with a strong crystal field. Using the chiral Eu(III) lumino-glass, the first application of CPL paint as a security ink (to display a sun or moon shape) are demonstrated. These glass-type chiral Eu(III) complexes are promising candidates for future devices with polarized light information.

## Results and discussion

### Luminescence intensity of Eu(III) lumino-glass

**Fig. 2:**
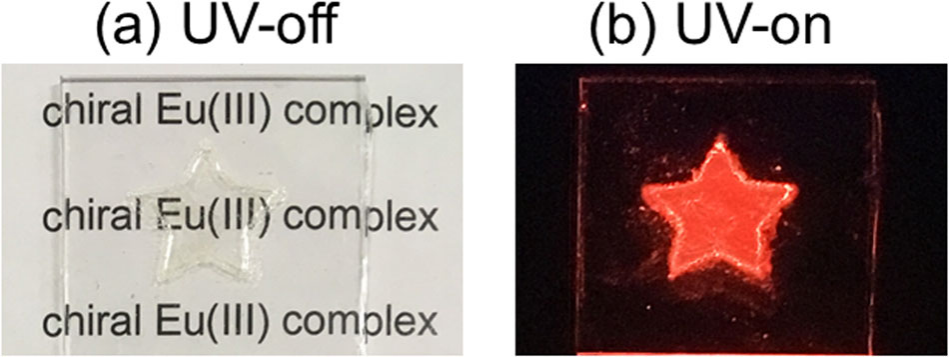
Photographs of Eu(III) lumino-glass. Photographs of Eu(III) lumino-glass (Eu(+tfc)-tmpo(2). UV-off (**a**), UV-on (**b**, *λ*_ex_ = 365 nm)).

**Fig. 3:**
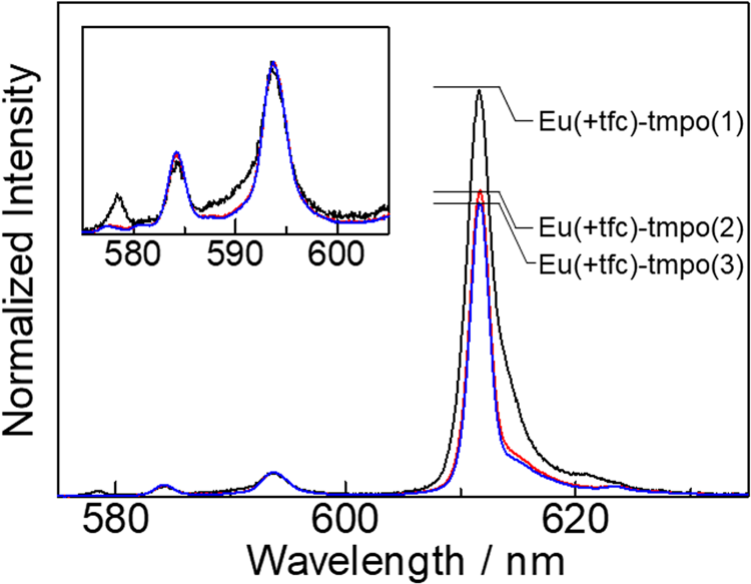
Emission spectra. Emission spectra of Eu(+tfc)-tmpo(1) (black line), Eu(+tfc)-tmpo(2) (red line), and Eu(+tfc)-tmpo(3) (blue line).

Emission properties of Eu(III) lumino-glass (Fig. 2) were evaluated using emission spectra and time-resolved emission measurement. The emission spectra of the Eu(+tfc)-tmpo(*n*) films (*n* = 1, 2, 3, *n*: number of equivalents relative to Eu(+tfc)_3_) are shown in Fig. 3 (Supplementary Note 1 and Supplementary Figs. 1–3). The spectra were normalized using the integrated intensity of the magnetic dipole transition (MD: ^5^D_0_ → ^7^F_1_). Two peaks were observed at 584 and 594 nm for the MD transition (Fig. 3, inset). The strong emission bands near 612 nm were attributed to the electric dipole transition (ED: ^5^D_0_ → ^7^F_2_). The emission band of Eu(+tfc)-tmpo(1) was different from those of Eu(+tfc)-tmpo(2) and Eu(+tfc)-tmpo(3). The luminescence intensity ratio of the ED/MD transition (A_ED_/A_MD_) decreased with increasing *n* (i.e., increasing number of tmpo molecules), indicating that the Eu(III) complex with tmpo ligands forms a symmetric coordination geometry^28^. The crystal field splitting in the MD transition was found to be 274 cm^−1^. Small A_ED_/A_MD_ and large crystal field splitting values were also observed in a comparative study with other Eu(III) complexes (Supplementary Note 2–3, Supplementary Figs. 4–7, and Supplementary Table 1). These results indicated that a symmetric coordination structure with a strong crystal field was formed by the tmpo ligands, which was attributed to the large steric hindrance and multiple interaction of tmpo ligands.

To obtain information regarding the coordination structure around the luminescent Eu(III) center, the time-resolved emission profiles of the complexes were measured. The emission lifetimes were estimated using triple exponential decays (Table 1), and were consistent with the existence of several metastable structures. The relative lifetime of the τ_3_ component increased with the addition of more equivalents of tmpo. The emission decay phenomenon of Eu(+tfc)-tmpo(2) was similar to that of Eu(+tfc)-tmpo(3). The emission quantum efficiencies excited by the tfc ligands (*Φ*_tot_) of the films are also shown in Table 1. The *Φ*_tot_ values also increased with the number of equivalents of tmpo added. The emission quantum yield of Eu(+tfc)-tmpo(2) (*Φ*_tot_ = 13 %) was much larger than that of previously reported Eu(III)-Cs(I) systems (*Φ*_tot_ < 1%) with a large *g*_CPL_ value (−1.38) in solution^24,25^.

**Table 1:** Photophysical properties of Eu(III) lumino-glass.

	*τ*_1_ (ms)^a^	*τ*_2_ (ms)^a^	*τ*_3_ (ms)^a^	*Φ*_tot_ (%)^b^
Eu(+tfc)-tmpo(1)	0.10 (11%)	0.28 (44%)	0.70 (45%)	2.6
Eu(+tfc)-tmpo(2)	0.14 (6%)	0.37 (34%)	0.74 (60%)	13
Eu(+tfc)-tmpo(3)	0.16 (5%)	0.41 (34%)	0.76 (61%)	13

^a^Emission lifetime (*λ*_ex_ = 356 nm, *λ*_em_ = 612 nm).

^b^*Φ*_tot_ (*λ*_ex_ = 370 nm).

### CPL properties of Eu(±tfc) with tmpo ligands

**Fig. 4:**
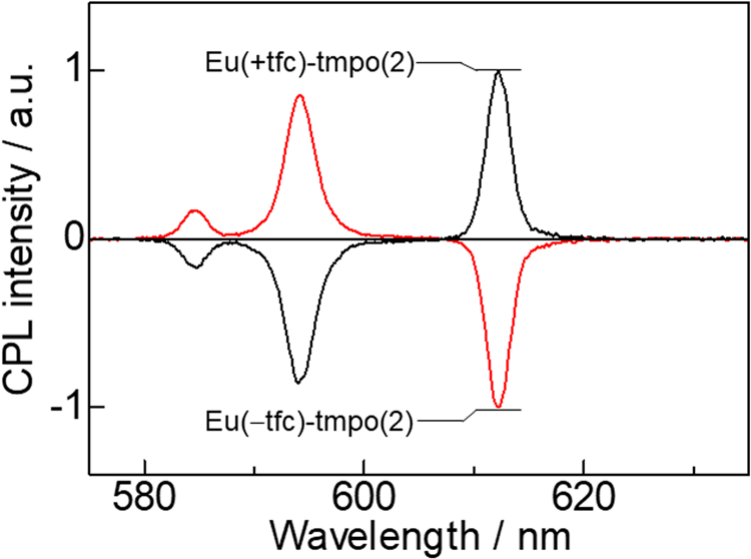
CPL spectra. CPL spectra of Eu(+tfc)-tmpo(2) (black line) and Eu(−tfc)-tmpo(2) (red line).

The CPL spectra of Eu(±tfc)-tmpo(2) are shown in Fig. 4 (Supplementary Note 4–6, Supplementary Figs. 8–15, and Supplementary Table 2). Large CPL signals were observed for the MD transition at 594 nm (Eu(+tfc)-tmpo(2): *g*_CPL_ = −1.2, Eu(−tfc)-tmpo(2): *g*_CPL_ = 1.2) and the ED transition at 612 nm (Eu(+tfc)-tmpo(2): *g*_CPL_ = 0.15, Eu(−tfc)-tmpo(2): *g*_CPL_ = −0.15). The *g*_CPL_ value in the MD transition is almost same as that of Eu(+tfc)-tmpo(1) (*g*_CPL_ = −1.0) and Eu(+tfc)-tmpo(3) (*g*_CPL_ = −1.2; Supplementary Note 7 and Supplementary Fig. 16). The CPL spectrum of Eu(+tfc) with two equivalents of tmpo in solution also exhibited a large CPL signal (*g*_CPL_ = −1.0) (Supplementary Notes 8 and 9, Supplementary Figs. 17–19, Supplementary Table 3, and Supplementary Data 1–3). The *g*_CPL_ values at 594 nm (film: *g*_CPL_ = −1.2, solution: *g*_CPL_ = −1.0) were as large as that previously reported (*g*_CPL_ = −1.38) for Eu(III)-Cs(I) systems^24,25^. These values were different from the small *g*_CPL_ value of a mononuclear Eu(III) complex with tightly coordinated phosphine oxide ligands (Eu(+tfc)_3_(tppo)_2_, tppo: triphenylphosphine oxide, *g*_CPL_ = 0.09) and a polynuclear Eu(III) complex ([Eu(+tfc)_3_dpbp]_*n*_, dpbp: 4,4-bis(diphenylphosphoryl)biphenyl, *g*_CPL_ = 0.17)^26^. The *g*_CPL_ value of Eu(+tfc)-tmpo(2) was also higher than those of the other Eu(III) lumino-glasses in a comparative study (Supplementary Fig. 7 and Supplementary Table 1). In the comparative compounds, the Eu(+tfc)-L1(2) showed a relatively large *g*_CPL_ signal. We attributed the large *g*_CPL_ value of the Eu(III) lumino-glass to the large J-mixing (J: total angular momentum)^22^ induced by the symmetric coordination geometry with a strong crystal field. In addition, the methoxy group in the ortho-position in the phosphine oxide ligands play an important role in the formation of the characteristic coordination field.

### Demonstration of a CPL application

**Fig. 5:**
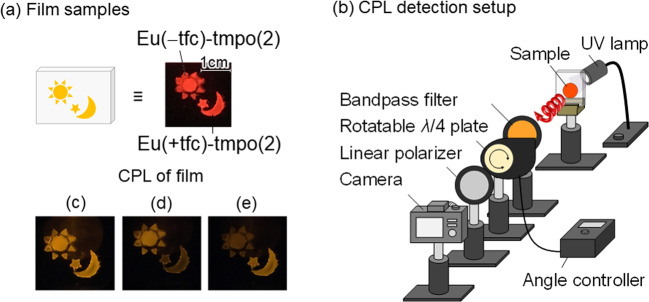
CPL detection. **a** Photographs and images of the films of Eu(+tfc)-tmpo(2) and Eu(−tfc)-tmpo(2) prepared on glasses under UV-light (*λ*_ex_ = 365 nm). **b** The simple setup for CPL detection. Photographs of the emission (*λ*_em_ = 594 nm) related to **c** left and right, **d** left, or **e** right circularly polarized light from the samples.

We also demonstrated a CPL paint application using the amorphous Eu(III) lumino-glass. The prepared samples are shown in Fig. 5a. Transparent glasses of Eu(+tfc)-tmpo(2) and its enantiomer (Eu(−tfc)-tmpo(2)) were prepared on a substrate using a casting method (Eu(+tfc)-tmpo(2): sun symbol; Eu(−tfc)-tmpo(2): moon symbol). The experimental setup for visualizing the CPL image is depicted in Fig. 5b. In the setup, the emission of the lumino-glasses is detected using a camera with a linear polarizer, a rotatable λ/4 plate, and a bandpass filter (594 nm). The left- or right-handed polarized light is detected by rotating the λ/4 plate 90° clockwise or anticlockwise using an angle controller. The photographs of the emission of the samples using total light (without the *λ*/4 plate), left-handed, and right-handed circularly polarized light are shown in Fig. 5c, d, and e, respectively. The total emission from the Eu(+) and Eu(−) lumino-glass is observed in photograph (Fig. 5c). Under clockwise and anticlockwise rotation, the sun and moon shapes of the Eu(+) and Eu(−) lumino-glasses were observed, respectively (photographs Fig. 5d, e). This is the first demonstration of naked-eye detection of a security paint based on the CPL phenomenon using amorphous Eu(III) lumino-glasses.

To the best of our knowledge, the first lanthanide lumino-glass with an extra-large CPL and a relatively high emission quantum yield was reported. We revealed that symmetric coordination with a strong crystal field is a key factor for the enhancement of *g*_CPL_. We also successfully demonstrated a security paint based on the CPL phenomenon using Eu(III) lumino-glass for the first time. Our results provide new insights into the design of Eu(III) complexes with excellent CPL performance and high emission intensity, which should lead to new applications of CPL materials as security inks.

## Methods

### Materials

Europium(III) acetate *n*-hydrate, acetone-d_6_ (99.9%) and 28% ammonia solution were purchased from Wako Pure Chemical Industries Ltd. Tris(2,4,6-trimethylphenyl)phosphine, (+)-3-(trifluoroacetyl)camphor and (−)-3-(trifluoroacetyl)camphor were purchased from Sigma-Aldrich Co. Tris(2,6-dimethoxyphenyl)phosphine, tris(4-methoxyphenyl)phosphine, and tris(4-methylphenyl)phosphine were purchased from Tokyo Chemical Industry Co., Ltd. All other chemicals and solvents were of reagent grade and were used without further purification.

### Apparatus

Electrospray ionization mass spectra (ESI-MS) were measured by using a Thermo Scientific Exactive instrument. Elemental analyses were performed on an Exeter Analytical CE440. Emission spectra and emission lifetimes were measured using a Horiba/Jobin-Yvon FluoroLog-3 spectrofluorometer. CPL spectra were measured using a JASCO CPL-300 spectrofluoropolarimeter. DSC measurements were recorded on a SII DSC 7020 heat flux meter. Proton nuclear magnetic resonance (^1^H NMR) spectra were recorded on an auto-NMR JEOL ECS 400 MHz.

### Synthesis of Tris(2,6-dimethoxyphenyl)phosphine oxide

Tris(2,6-dimethoxyphenyl)phosphine (8.84 g, 0.02 mol) was dissolved in dichloromethane (30 mL) in a 100-mL flask, which was cooled in an ice bath. H_2_O_2_ solution (5 mL) was added slowly to the solution. The mixture was stirred for 3 h. The product was extracted with dichloromethane and saturated NaCl aqueous solution. The organic layer was dried with anhydrous MgSO_4_, and the solvent was evaporated. The obtained oil was precipitated with acetone and hexane. The precipitate was filtered and washed with acetone to afford white powder.

Yield: 3.95 g (41%). *δ*/ppm = 7.23 (t, 3H, *J* = 8 Hz, Ar), 6.48 (dd, 6H, *J* = 8 and 4.8 Hz, Ar), 3.51 (s, 18H, CH_3_). ESI-MS (m/z): [M+H]^+^ calculated for C_24_H_28_O_7_P, 459.15; found, 459.16. Elemental analysis: Calcd for C_24_H_27_O_7_P: C, 62.88%, H, 5.94%. Found: C, 62.55%, H, 5.88%.

### Synthesis of Tris(3-trifluoroacetyl-(+)-camphorato)europium(III)

Europium(III) acetate *n*-hydrate (0.36 g) was dissolved in distilled water (150 mL). (+)-3-triflouroacetyl camphor (+tfc, 0.50 g, 2.0 mmol) in methanol (20 mL) was added to the solution. A few drops of 28% ammonia solution were added and the mixture was stirred for 3 h at room temperature. The obtained powder was washed with distilled water to afford yellow powder.

Yield: 0.42 g (68%). Elemental analysis: Calcd for C_36_H_46_EuF_9_O_8_: C, 46.51%, H, 4.99%. Found: C, 46.45%, H, 4.91%.

### Synthesis of Tris(3-trifluoroacetyl-(−)-camphorato)europium(III)

[Eu(−tfc)_3_(H_2_O)_2_] was prepared using the same method for [Eu(+tfc)_3_(H_2_O)_2_], starting from (−)-3-triflouroacetyl camphor, yielding yellow powder.

### Preparation of Eu(III) lumino-glass

Eu(±tfc)_3_(H_2_O)_2_ (3 mg, 0.003 mmol) and *n* equivalents of tmpo (1.5 mg, 3.0 mg, or 4.5 mg for *n* = 1, 2, or 3, respectively) were dissolved in dichloromethane (0.1 mL) (Supplementary Note 1 and Supplementary Fig. 1). The solution was casted onto a glass substrate and allowed to slowly evaporate, yielding a transparent film of the Eu(+tfc)-tmpo(*n*) (*n* = 1, 2, 3, *n*: number of equivalents relative to Eu(+tfc)_3_). The obtained film was characterized by ESI-MS, XRD (Supplementary Fig. 2), and DSC (Supplementary Fig. 3). ESI mass spectrometry revealed the existence of the Eu(III) complex [Eu(+tfc)_2_(tmpo)_2_]^+^ (cacld: 1563.41, found: 1563.38). The XRD signals of the transparent film were broad, similar to those of typical amorphous organic molecules. In the DSC curve, a characteristic endothermic peak with shoulders indicating a glass transition was observed at ~50 °C (Supplementary Fig. 3). The glass-typed Eu(III) complexes showed bright red luminescence (“Eu(III) lumino-glass”, ex. Eu(+tfc)-tmpo(2), Fig. 2).

## Supplementary information

Supplementary Information

Description of Additional Supplementary Files

Supplementary Data 1

Supplementary Data 2

Supplementary Data 3

## Data Availability

The authors declare that the data supporting the findings of this study are available within the paper and its supplementary information.
