# Supplementary material for: Chiral lanthanide lumino-glass for a circularly polarized light security device
Source: Commun Chem. 2020 Aug 25;3:119. doi: 10.1038/s42004-020-00366-1 (PMC9814105; doi:10.1038/s42004-020-00366-1)
Supplement: Supplementary file 1 — Supplementary Information [file 42004_2020_366_MOESM1_ESM.pdf]

Supporting Information

Chiral lanthanide lumino-glass for a circularly polarized light security device

Yuichi Kitagawa#[1,2*], Satoshi Wada#[1], MD J. Islam[2], Kenichiro Saita[3], Masayuki Gon[4], Koji Fushimi[1], Kazuo Tanaka[4], Satoshi Maeda[2,3] & Yasuchika Hasegawa[1,4*]

[1]Faculty of Engineering, Hokkaido University, N13 W8, Kita-ku, Sapporo, Hokkaido 060–8628, Japan.

[2]Institute for Chemical Reaction Design and Discovery (WPI-ICReDD), Hokkaido University, N21 W10, Sapporo, Hokkaido 001-0021, Japan.

[3]Department of Chemistry, Faculty of Science, Hokkaido University, Sapporo 060-0810, Japan.

[4]Graduate School of Engineering, Kyoto University, Katsura, Nishikyo-ku, Kyoto 615-8510, Japan.

#These authors contributed equally.

Correspondence and requests for materials should be addressed to Y.K. (email: y-kitagawa@eng.hokudai.ac.jp) or Y.H. (email: hasegaway@eng.hokudai.ac.jp)

Supplementary Methods

Synthesis of Tris(2-methoxyphenyl)phosphine oxide (L1)[1]

Tris(2-methoxyphenyl)phosphine (2.0 g, 5.7 mmol) was dissolved in dichloromethane (50 mL) in a 100 mL flask. The solution was cooled in an ice bath and H_2O_2 solution (5 mL) was added slowly to the solution. The mixture was stirred for 3 h. The product was extracted with dichloromethane and saturated NaCl aqueous solution. The organic layer was dried with anhydrous $MgSO_4$, and the solvent was evaporated. The solid powder was washed with ethyl acetate to afford white powder.

Yield: 1.8 g (86%). ^{1}H NMR (400 MHz, $CDCl_3$) δ/ppm = 7.55–7.41 (m, 6H, Ar), 7.02–6.93 (m, 3H, Ar), 6.92–6.87 (m, 3H, Ar), 3.57 (s, 9H, CH_3).

Synthesis of Tris(3-methoxyphenyl)phosphine oxide (L2)[1]

Tris(3-methoxyphenyl)phosphine (0.5 g, 0.14 mmol) was dissolved in dichloromethane (20 mL) in a 100 mL flask. The solution was cooled in an ice bath and H_2O_2 solution (5 mL) was added slowly to the solution. The mixture was stirred for 3 h. The product was extracted with dichloromethane and saturated NaCl aqueous solution. The organic layer was dried with anhydrous $MgSO_4$, and the solvent was evaporated. The precipitate was recrystallized by methanol and ethylacetate.

Yield: 0.42 g (82 %). ^{1}H NMR (400 MHz, $CDCl_3$) δ/ppm = 7.40–7.24 (m, 6H, Ar), 7.18–7.04 (m, 6H, Ar), 3.80 (s, 9H, CH_3).

Synthesis of Tris(4-methoxyphenyl)phosphine oxide (L3)[2]

Tris(4-methoxyphenyl)phosphine (1.0 g, 0.28 mmol) was dissolved in dichloromethane (30 mL) in a 100 mL flask. The solution was cooled in an ice bath and H_2O_2 solution (5 mL) was added slowly to the solution. The mixture was stirred for 3 h. The product was extracted with dichloromethane and saturated NaCl aqueous solution. The organic layer was dried with anhydrous $MgSO_4$, and the solvent

was evaporated. The obtained oil was precipitated with acetone and hexane. The precipitate was filtered and washed with acetone to afford white powder.

Yield: 0.55 g (53%). ^{1}H NMR (400 MHz, CD_3COCD_3) δ/ppm = 7.62–7.49 (m, 6H, Ar), 7.12–6.97 (m, 6H, Ar), 3.85 (s, 9H, CH_3).

Synthesis of Tris(4-methylphenyl)phosphine oxide (L4)[2]

Tris(4-methylphenyl)phosphine (1.0 g, 3.3 mmol) was dissolved in dichloromethane (30 mL) in a 100 mL flask. The solution was cooled in an ice bath and H_2O_2 solution (5 mL) was added slowly to the solution. The mixture was stirred for 3 h. The product was extracted with dichloromethane and saturated NaCl aqueous solution. The organic layer was dried with anhydrous $MgSO_4$, and the solvent was evaporated. The obtained oil was precipitated with acetone and hexane. The precipitate was filtered and washed with acetone to afford white powder.

Yield: 0.90 g (86%). ^{1}H NMR (400 MHz, CD_3COCD_3) δ/ppm = 7.58–7.47 (m, 6H, Ar), 7.37–7.27 (m, 6H, Ar), 2.38 (s, 9H, CH_3).

Synthesis of Tris(2,4,6-trimethylphenyl) phosphine oxide (L5)[3]

Tris(2,4,6-trimethylphenyl)phosphine (0.350 g, 0.9 mmol) was dissolved in dichloromethane (30 mL) in a 100 mL flask. The solution was cooled in an ice bath and H_2O_2 solution (5 mL) was added slowly to the solution. The mixture was stirred for 3 h. The product was extracted with dichloromethane and saturated NaCl aqueous solution. The organic layer was dried with anhydrous $MgSO_4$, and the solvent was evaporated. The obtained oil was precipitated with acetone and hexane. The precipitate was filtered and washed with acetone to afford white powder.

Yield: 0.16 g (44%).^{1}H NMR (400 MHz, $C_2D_2Cl_4$) δ/ppm = 6.83 (br, 6H), 2.29 (s, 9H, CH_3), 2.17 (br, 18H, CH_3).

Supplementary Note 1

The NMR spectra of the mixed solution of $Eu(tfc)_3(H_2O)_2$ (1 mM) and tmpo (0, 1, 2, 3 equivalents) were measured (Supplementary Figure 1). The low magnetic field region in the NMR signal indicates the existence of tmpo in the solution. In addition, the tmpo ligands had two kinds of proton, which indicated that the existence of several conformation in CD_2Cl_2.

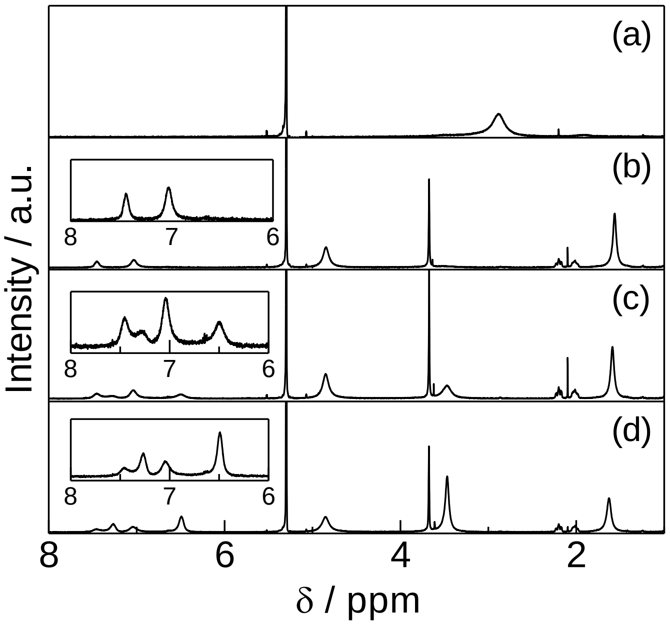

Supplementary Figure 1. NMR spectra of $Eu(+tfc)_3(H_2O)_2$ (1 mM) with 0 (a), 1 (b), 2 (c), and 3 (d) equivalents of tmpo ligand in CD_2Cl_2.

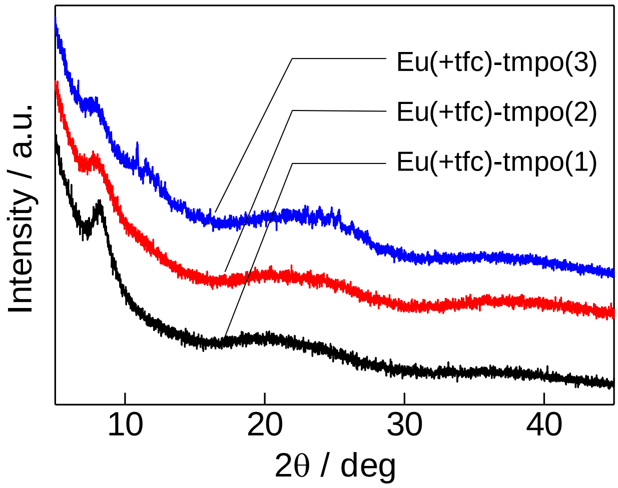

Supplementary Figure 2. XRD patterns of Eu(+tfc)-tmpo(1), Eu(+tfc)-tmpo(2), and Eu(+tfc)-tmpo(3).

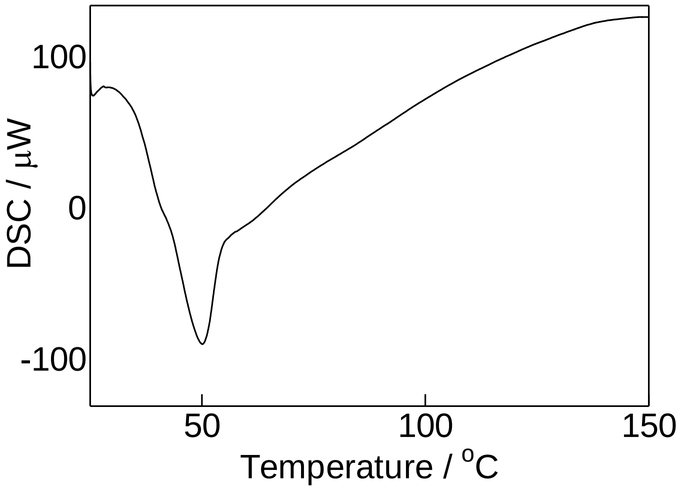

Supplementary Figure 3. DSC pattern of Eu(+tfc)-tmpo(2).

Supplementary Note 2

To understand the factors crucial to the construction of an amorphous Eu(III) structure, five phosphine oxide ligands (Figure 1) were prepared. Eu(+tfc)$_3$(H_2O)$_2$ (3 mg, 0.003 mmol) and 2 equivalents of L1 (2.4 mg), L2 (2.4 mg), L3 (2.4 mg), L4 (2.2 mg), and L5 (2.8 mg) were dissolved in dichloromethane (0.1 mL). The solutions were cast onto a glass substrate and allowed to evaporate, yielding the Eu(III) complexes Eu(+tfc)-L1(2), Eu(+tfc)-L2(2), Eu(+tfc)-L3(2), Eu(+tfc)-L4(2), and Eu(+tfc)-L5(2) in solid form. The XRD signals of the films exhibited broad peaks (Supplementary Figure 4), which indicated the low-crystalline state. The results indicate that Eu(tfc)$_3$ complexes with phosphine oxide-type ligands tend to form amorphous structures, although the crystal structures of Eu(tfc) with triphenylphosphine oxide and 4,4-bis(diphenylphosphoryl)biphenyl have been obtained previously.[4]

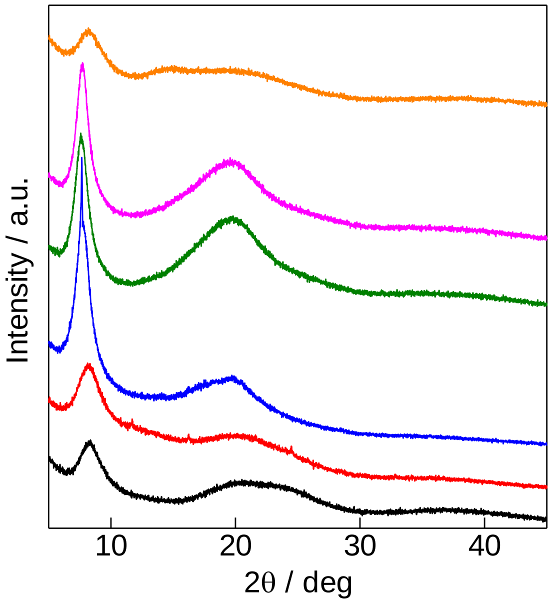

Supplementary Figure 4. XRD spectral patterns of Eu(+tfc)-tmpo(2) (black line), Eu(+tfc)-L1(2) (red line), Eu(+tfc)-L2(2) (blue line), Eu(+tfc)-L3(2) (green line), Eu(+tfc)-L4(2) (pink line), Eu(+tfc)-L5(2) (orange line).

Supplementary Note 3

The emission spectra of the $^5D_0 \rightarrow ^7F_1$ transitions of Eu(+tfc)-tmpo(2), Eu(+tfc)-L1(2), Eu(+tfc)-L2(2), , Eu(+tfc)-L3(2), Eu(+tfc)-L4(2), and Eu(+tfc)-L5(2) are shown in Supplementary Figure 5. The $^7F_1$ energy level of Eu(III) ions in a typical eight-coordinate structure (C_{4v} or D_{2d}) splits into two Stark sublevels.[5] The two bands at 584 and 593 nm in the CPL spectra were assigned to the $A_1 \rightarrow A_2$ and $A_1 \rightarrow E$ transitions, respectively. The Stark splitting energies (ΔE_{ss}) of Eu(+tfc)-tmpo(2), Eu(+tfc)-L1(2), Eu(+tfc)-L2(2), , Eu(+tfc)-L3(2), Eu(+tfc)-L4(2), and Eu(+tfc)-L5(2) were estimated to be 274 cm^{-1}, 269 cm^{-1}, 138 cm^{-1}, 126 cm^{-1}, 152 cm^{-1}, and 249 cm^{-1} respectively.

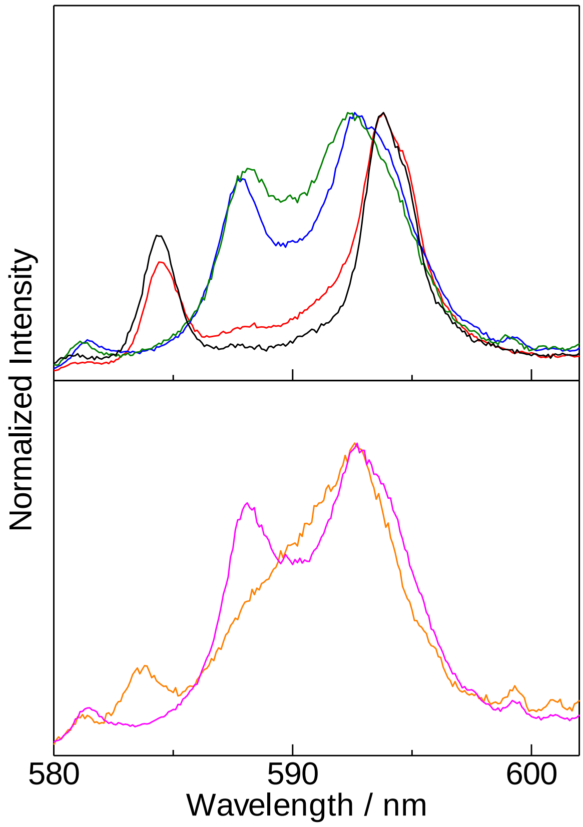

Supplementary Figure 5. Emission spectra ($^5D_0 \rightarrow ^7F_1$ transition) of Eu(+tfc)-tmpo(2) (black line), Eu(+tfc)-L1(2) (red line), Eu(+tfc)-L2(2) (blue line), Eu(+tfc)-L3(2) (green line), Eu(+tfc)-L4(2) (pink line), and Eu(+tfc)-L5(2) (orange line).

For Eu(III) complexes, it is well-known that there is a photophysical relationship between the crystal field symmetry and the emission intensity ratio $^5D_0 \rightarrow ^7F_2/^5D_0 \rightarrow ^7F_1$ (A_{ED}/A_{MD}).[4] The emission bands for the $^5D_0 \rightarrow ^7F_2$ transitions of Eu(+tfc)-tmpo(2), Eu(+tfc)-L1(2), Eu(+tfc)-L2(2), Eu(+tfc)-L3(2), Eu(+tfc)-L4(2), and Eu(+tfc)-L5(2) are also shown in Supplementary Figure 6. The A_{ED}/A_{MD} values of Eu(+tfc)-tmpo(2), Eu(+tfc)-L1(2), Eu(+tfc)-L2(2), Eu(+tfc)-L3(2), Eu(+tfc)-L4(2), and Eu(+tfc)-L5(2) were estimated to be 6.5, 10.6, 17.3, 16.7, 17.6 and 18.1, respectively. The FWHM of Eu(+tfc)-tmpo(2), Eu(+tfc)-L1(2), Eu(+tfc)-L2(2), Eu(+tfc)-L3(2), Eu(+tfc)-L4(2), and Eu(+tfc)-L5(2) were estimated to be 46 cm^{-1}, 67 cm^{-1}, 202 cm^{-1}, 202 cm^{-1}, 199 cm^{-1}, and 88 cm^{-1}, respectively.

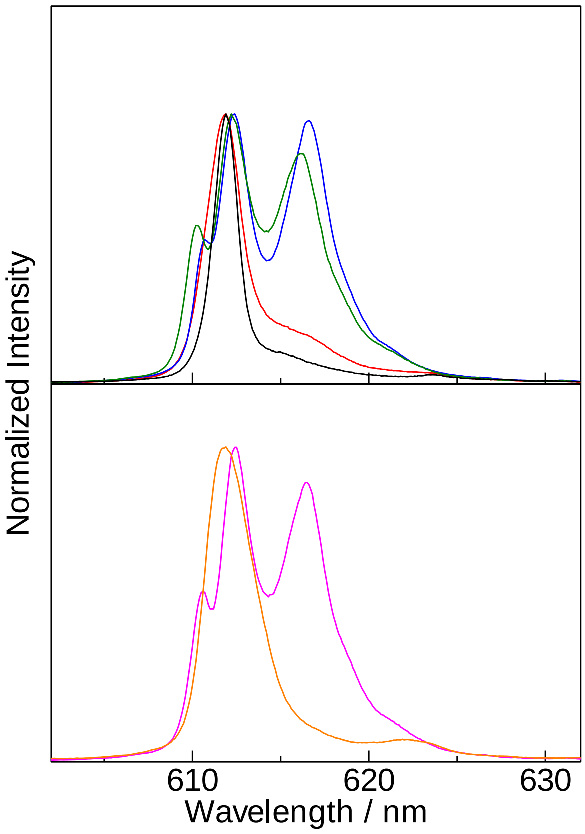

Supplementary Figure 6. Emission spectra ($^5D_0 \rightarrow ^7F_2$ transition) of Eu(+tfc)-tmpo(2) (black line), Eu(+tfc)-L1(2) (red line), Eu(+tfc)-L2(2) (blue line), Eu(+tfc)-L3(2) (green line), Eu(+tfc)-L4(2) (pink line), and Eu(+tfc)-L5(2) (orange line).

The CPL spectra of Eu(+tfc)-tmpo(2), Eu(+tfc)-L1(2), Eu(+tfc)-L2(2), Eu(+tfc)-L3(2), Eu(+tfc)-L4(2), and Eu(+tfc)-L5(2) are shown in Supplementary Figure 7. The g_{CPL} values of Eu(+tfc)-L1(2), Eu(+tfc)-L2(2), Eu(+tfc)-L3(2), Eu(+tfc)-L4(2), and Eu(+tfc)-L5(2) were estimated to be −1.2, −1.0, 0.04, 0.07, 0.09, and −0.29, respectively.

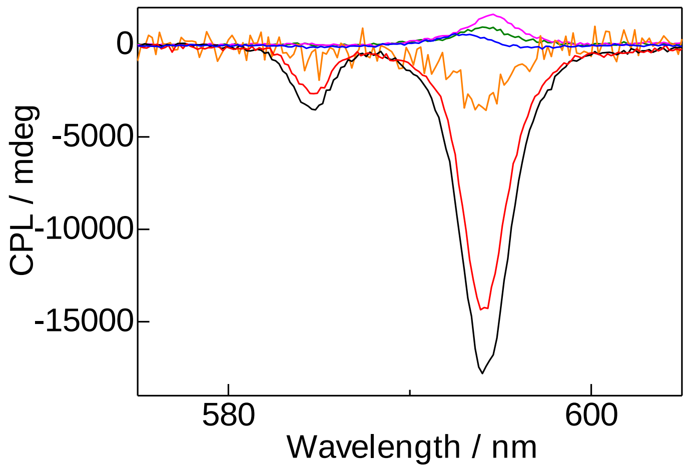

Supplementary Figure 7. CPL spectra of Eu(+tfc)-tmpo(2) (black line), Eu(+tfc)-L1(2) (red line), Eu(+tfc)-L2(2) (blue line), Eu(+tfc)-L3(2) (green line), Eu(+tfc)-L4(2) (pink line), and Eu(+tfc)-L5(2) (orange line).

Based on the results (Supplementary Table 1), we found that the Eu(III) complex with symmetric and strong crystal field show large g_{CPL} values. Herein, we compared the photophysical properties of the Eu(III) complex with those of the previous chiral Eu(III) complexes. Recently, we observed a large g_{CPL} value (= −1.0) of Eu(+tfc)$_3$(H$_2$O)$_2$ in acetone solution (Eu(+tfc)-acetone) without the presence of phosphine oxide ligands.[6] In addition, the g_{CPL} value, the photophysical parameter (ΔE_{SS}, FHWM), and the A_{ED}/A_{MD} were similar to those of the Eu(+tfc)-L1(2) (Supplementary Table 1). Previously, Muller observed an exceptionally large g_{CPL} value in an Eu(III)-Cs(I) system with characteristic chiral β-diketonate ligands containing a camphor framework (tetrakis(3-heptafluorobutylryl-(+)-

camphorato) (g_{CPL} = −1.38).[7] The compounds also showed a large ΔE_{SS} (Figure 2 in Ref 7), and a narrow FWHM (Figure 2 in Ref 7).

Supplementary Table 1. Summary of photophysical results.

Compounds	ΔE_{SS} / cm^{-1}	FWHM / cm^{-1}	A_{ED}/A_{MD}	g_{CPL}
Eu(+tfc)-tmpo(2)	274	46	6.5	−1.2
Eu(+tfc)-L1(2)	269	67	10.6	−1.0
Eu(+tfc)-L2(2)	138	202	17.3	0.04
Eu(+tfc)-L3(2)	126	202	16.7	0.07
Eu(+tfc)-L4(2)	152	199	17.6	0.09
Eu(+tfc)-L5(2)	249	88	18.1	−0.29
Eu(+tfc)-acetone[6]	268	67	12.6	−1.0

Supplementary Note 4

We measured the g_{CPL} value depending on the rotation angle along the light axis, and also measured the g_{CPL} value depending on the rotation angle along the light axis by reversing the glass containing the Eu(III) film. The g_{CPL} values were almost similar in both cases (Supplementary Figure 8).

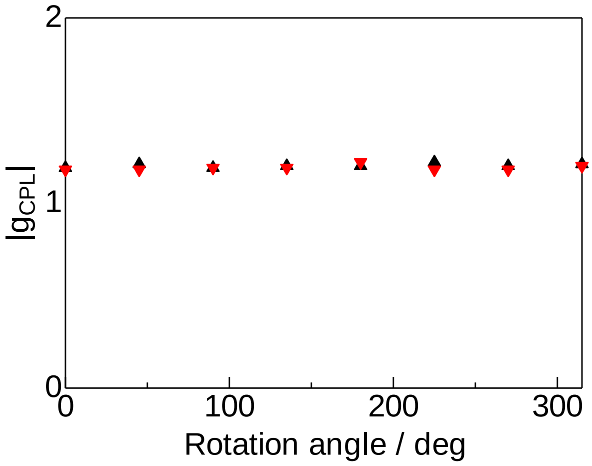

Supplementary Figure 8. g_{CPL} spectra of Eu(+tfc)-tmpo(2) depending on the rotation angle along the light axis (Black triangle: coated surface is excitation side, Red inverted triangle: coated surface is detection side).

Supplementary Note 5

We prepared four films using the spin-coater as follows:

Preparation of film-1: First, the Eu(+tfc)$_3$(H_2O)$_2$ (3 mg) and tmpo (3 mg) were dissolved into CH_2Cl_2 (0.5 mL) using a vortex, after which the resulting solution was filtered. After filtration, the film on the quartz glass was prepared using 200 μl of the solution and a spin-coater (1000 rpm, 30 sec), after which the film was dried under reduced pressure for 4 h.

Preparation of film-2: First, the Eu(+tfc)$_3$(H_2O)$_2$ (3 mg) and tmpo (3 mg) were dissolved into CH_2Cl_2 (0.5 mL) using a vortex, after which the resulting solution was filtered. After filtration, the film on quartz glass is prepared using 200 μl solution and spin-coater (500 rpm, 30 sec), and drying under reduced pressure for 4h.

Preparation of film-3: First, the Eu(+tfc)$_3$(H_2O)$_2$ (30 mg) and tmpo (30 mg) were dissolved into CH_2Cl_2 (5 mL) using a vortex, after which the resulting solution was filtered. After filtration, the film on quartz glass is prepared using 200 μl solution and spin-coater (1000 rpm, 30 sec), and drying under reduced pressure for 4h.

Preparation of film-4: First, the Eu(+tfc)$_3$(H_2O)$_2$ (30 mg) and tmpo (30 mg) were dissolved into CH_2Cl_2 (5 mL) using a vortex, after which the resulting solution was filtered. After filtration, the film is prepared using 200 μl solution and spin-coater (1000 rpm, 30 sec), and drying under reduced pressure for 4h.

The electronic absorption spectra of the films are shown in Supplementary Figure 9. The thickness was roughly estimated by absorbance at 350 nm. The estimated thickness of the spin-coat film-1, 2, 3, and 4 were 140, 170, 150, and 130 nm, respectively, which were much lower than those of the cast films (2340 nm). The emission spectra are shown in Supplementary Figure 10. The emission spectral shapes of the spin-coat films (ΔE_{ss}: 269 cm^{-1}, FWHM: 43 cm^{-1}, A_{ED}/A_{MD}: 6.8 cm^{-1}) were similar to

those of the cast films, indicating the similar coordination geometry of the spin-coat films and the cast films. In contrast, the emission lifetime of the spin-coat film (τ_{ave} = 0.67-0.68 ms, Supplementary Table 2) was longer than that of the cast film (τ_{ave} = 0.61 ms). In addition, the emission quantum yield of the spin-coat film (Φ_{tot} = 14-16 %) was a little higher than that of the cast film (Φ_{tot} = 13 %). These results indicated that the photophysical properties of the film might be affected by the morphology of the film. Supplementary Figure 11 shows the CPL spectra of the spin-coat films. The estimated g_{CPL} value of the spin-coat film was higher than that of the cast film.

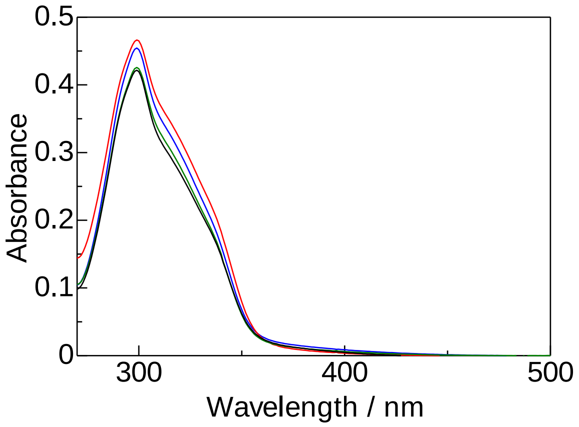

Supplementary Figure 9. Electronic absorption spectra of film-1 (black line), 2 (red line), 3 (blue line), and 4 (green line).

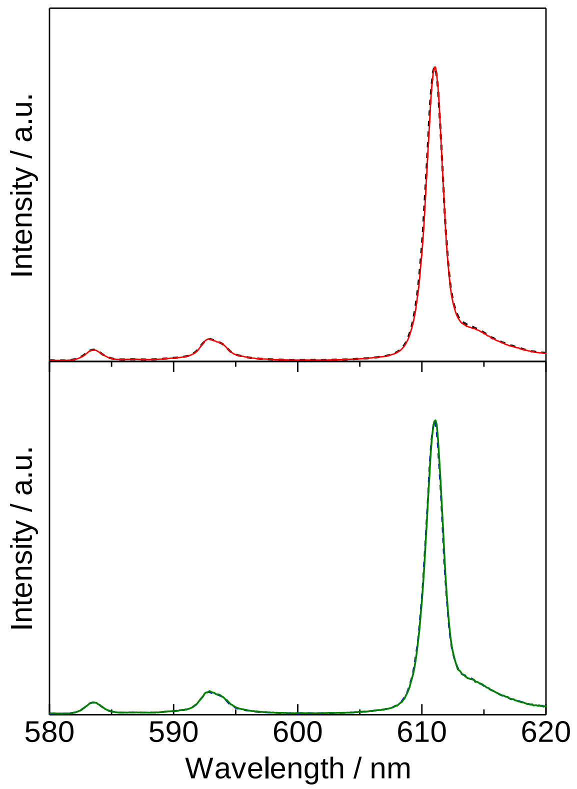

Supplementary Figure 10. Emission spectra (λ_{ex} = 350 nm) of film-1 (black broken line), 2 (red line), 3 (blue broken line), and 4 (green line).

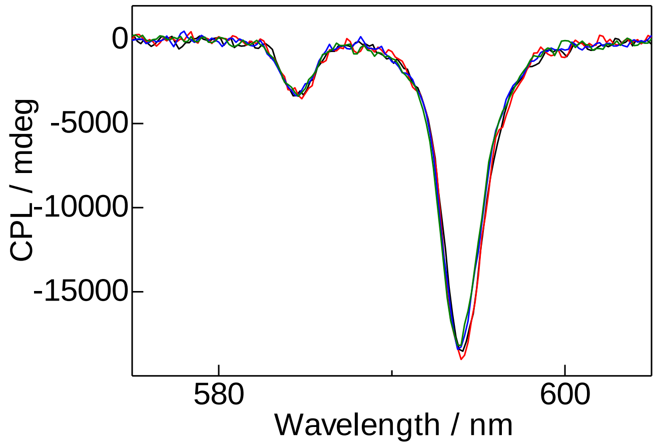

Supplementary Figure 11. CPL spectra (λ_{ex} = 350 nm) of film-1 (black line), 2 (red line), 3 (blue broken line), and 4 (green line). Normalized by DC.

Supplementary Table 2. Photophysical properties of film-1, 2, 3, and 4.

Compounds	τ_{ave} / ms	Φ_{tot} / %	g_{CPL}
**Film-1**	0.67	15	−1.3
**Film-2**	0.67	16	−1.3
**Film-3**	0.67	15	−1.3
**Film-4**	0.68	14	−1.3

Supplementary Note 6

We performed two kinds of stability test on the Eu(+tfc)-tmpo(2) films produced by cast method. First, we checked the air sensitivity, and exposed the sample to air atmosphere for one week. Then, we measured the emission spectrum and emission lifetime of the Eu(III) ion (Supplementary Figure 12-13). No visible changes were observed in the emission spectra and emission lifetime.

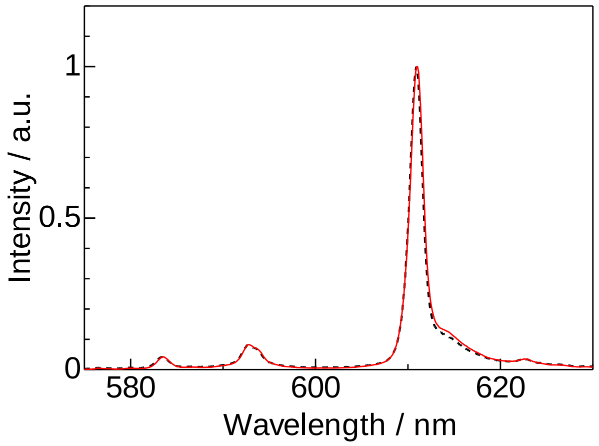

Supplementary Figure 12. Emission spectra (λ_{ex} = 350 nm) of Eu(+tfc)-tmpo(2) (black broken line), and one week after exposure to air atmosphere (red line).

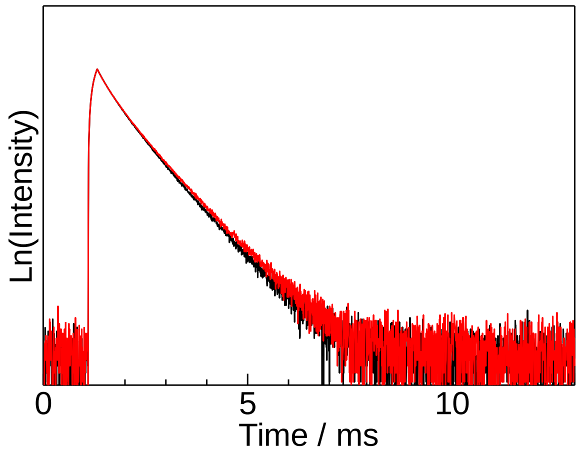

Supplementary Figure 13. Emission lifetimes (λ_{ex} = 356 nm) of Eu(+tfc)-tmpo(2) (black line), and one week after exposure to air atmosphere.

In addition, we observed the photostability of the Eu(+tfc)-tmpo(2) films under UV irradiation using UV crosslinker (300 mJ/cm^2, CL, 1000L, Funakoshi). No visible change was observed in the emission spectral and emission lifetime (Supplementary Figure 14-15).

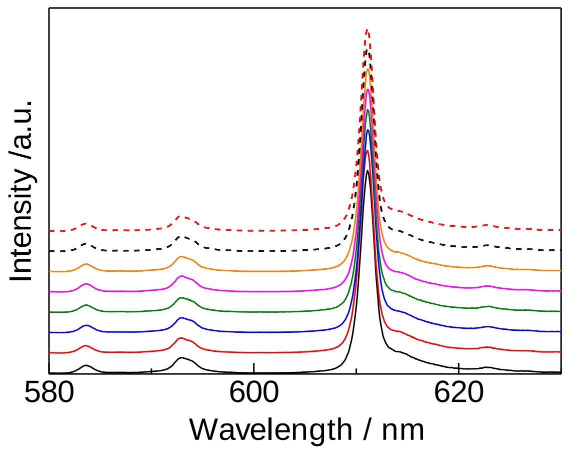

Supplementary Figure 14. Emission spectra (λ_{ex} = 350 nm) of Eu(+tfc)-tmpo(2) films after exposure to UV irradiation (black line: 0 h, red line: 1 h, blue line: 2 h, green line: 3 h, pink line: 4 h, orange line; 5 h, black broken line: 6h, and red broken line: 7 h).

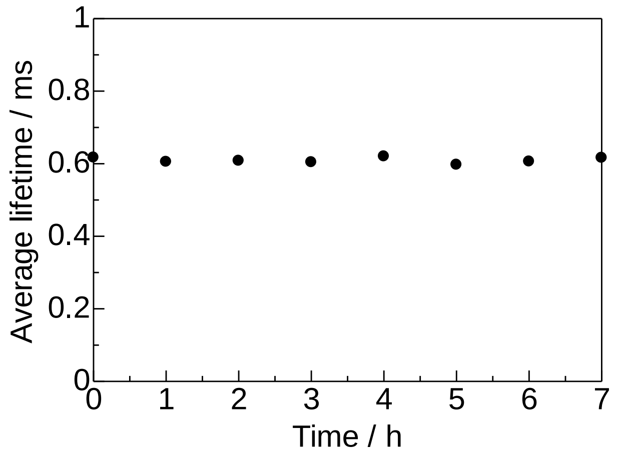

Supplementary Figure 15. Averaged emission lifetimes (λ_{ex} = 356 nm) of Eu(+tfc)-tmpo(2) films after exposure to UV irradiation.

Supplementary Note 7

The CPL spectra of Eu(+tfc)-tmpo(1) and Eu(+tfc)-tmpo(3) films are shown in Supplementary Figure 16. The g_{CPL} values are estimated to be –1.0 and –1.2, respectively.

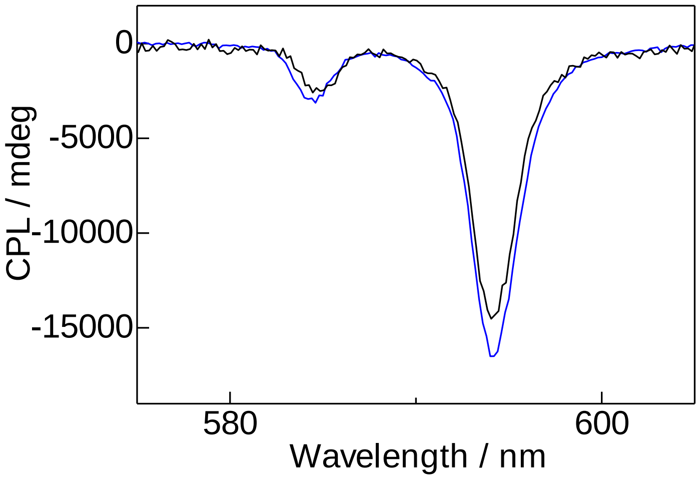

Supplementary Figure 16. CPL spectra of Eu(+tfc)-tmpo(1) (black line) and Eu(+tfc)-tmpo(3) (blue line). Normalized by DC voltage.

Supplementary Note 8

We evaluated the emission spectrum (Supplementary Figure 17), emission lifetime, and CPL spectrum (Supplementary Figure 18) of the solution containing Eu(tfc)$_3$(H_2O)$_2$ and tmpo ligands (toluene, Eu(tfc)$_3$(H_2O)$_2$: 1.0×10^{-3} M, tmpo 1.0×10^{-3} M). The presence of tmpo ligands in the solution was expected to induce several coordination geometry around Eu(III) ion. In addition, we also measured the emission properties of Eu(tfc)$_3$(H_2O)$_2$. We confirmed that the existence of the tmpo ligands induced a strong crystal field splitting in the $^5D_0 \rightarrow {}^7F_1$ transition, a small A_{ED}/A_{MD}, and a long emission lifetime (Supplementary Table 3). The long emission lifetime indicates the suppression of the vibrational quenching of H_2O, which indicates the coordination by the tmpo ligand. However, the emission quantum yield was low ($\Phi_{tot} < 1.0$ %), and the g_{CPL} values of the solution containing Eu(+tfc)$_3$(H_2O)$_2$ and tmpo ligands was estimated to be −1.0. The low emission quantum yield and the weaker g_{CPL} than that of the Eu(tfc)-tmpo(2) might be caused by the existence of the component with a short emission lifetime.

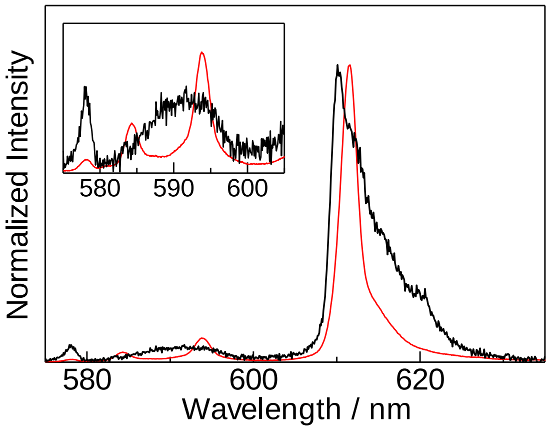

Supplementary Figure 17. Emission spectra of Eu(+tfc)(H_2O)$_2$ (black line) and Eu(+tfc)-tmpo(2) (red line) in toluene.

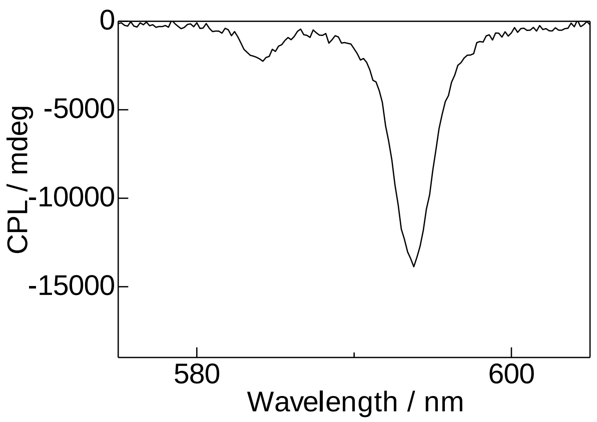

Supplementary Figure 18. CPL spectrum of Eu(+tfc)-tmpo(2) in toluene solution (1.0×10^{-3} M). Normalized by DC voltage.

Supplementary Table 3. Photophysical properties in toluene.

Compounds	ΔE_{SS} / cm^{-1}	FWHM / cm^{-1}	A_{ED}/A_{MD}	τ	g_{CPL}
Eu(+tfc)$_3$(H_2O)$_2$	143	134	10.2	0.11 (33 %), 0.02 (49 %), 0.006 (18%)	-[a]
Eu(+tfc)-tmpo(2)	277	67	8.2	0.47 (96 %), 0.01 (4 %)	−1.0

a: We cannot estimate the g_{CPL} because of the very weak signal.

Supplementary Note 9

At first, we explored structures of Eu(+tfc)$_3$(H_2O)(tmpo) and Eu(+tfc)$_3$(tmpo)$_2$ systematically to discuss the stabilities of complexes coordinated by the tmpo ligand. In this procedure, electronic structure calculations and automated structure searches were done by the GFN2-xTB method[8] implemented in the Orca program[9] and the SC-AFIR method[10] implemented in the GRRM program[11], respectively. As a result, 139 and 39 local minimum structures were obtained, respectively. The reason why the number of structures for Eu(+tfc)$_3$(tmpo)$_2$ was small would be because the tmpo ligand is bulky and the structural fractionality around the Eu center is small when two of them coexist. Important structures were further reoptimized at the ωB97X-D//BP86-D3/Def2-SV(P) level of the Gaussian 16 program[12]. Supplementary Figure 19 shows (a) the most stable Eu(+tfc)$_3$(H_2O)(tmpo) structure, (b) the most stable Eu(+tfc)$_3$(tmpo)$_2$ structure, and (c) the second most stable Eu(+tfc)$_3$(tmpo)$_2$ structure. Their relative free energy values are presented below these structures, where vibrational frequencies ν_i for modes having ν_i smaller than 50 cm^{-1} were set as 50 cm^{-1} in the vibrational entropy calculations. We note that these are gas-phase calculations and not used for quantitative comparisons with experimental data. Nevertheless, these results provided qualitative interpretations for experimental results as discussed below.

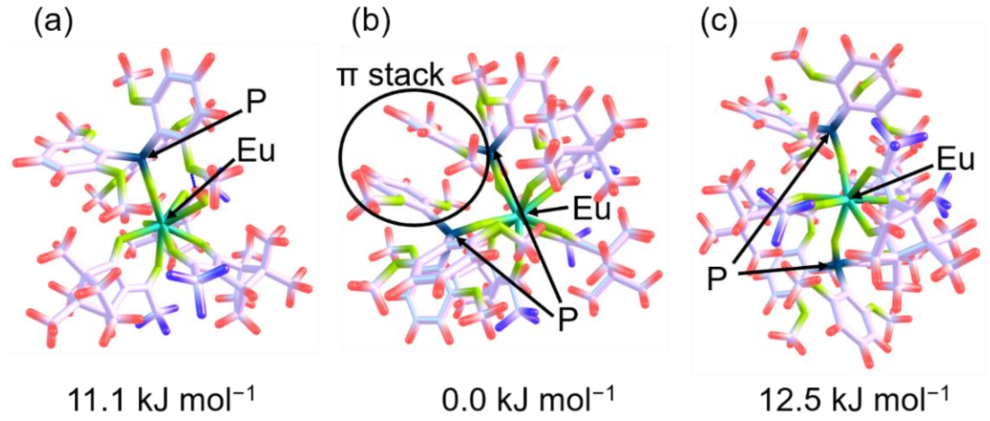

Supplementary Figure 19. (a) The most stable Eu(+tfc)$_3$(H_2O)(tmpo) structure (Supplementary Data 1), (b) the most stable Eu(+tfc)$_3$(tmpo)$_2$ structure (Supplementary Data 2), and (c) the second most stable Eu(+tfc)$_3$(tmpo)$_2$ structure (Supplementary Data 3). Relative free energy at room temperature is shown below each structure.

These results show that Eu(+tfc)$_3$(tmpo)$_2$ is more stable than Eu(+tfc)$_3$(H_2O)(tmpo). This suggests that both of the two H_2O molecules are likely substituted when the same amount of tmpo ligand is added, and this is consistent to the fact that there is only a small difference between experimental spectra obtained with two and three equivalent tmpo ligand. On the other hand, the energy gap between Eu(+tfc)$_3$(tmpo)$_2$ and Eu(+tfc)$_3$(H_2O)(tmpo) are only 11 kJ mol^{-1}. This suggests that a small amount (~1% with the Boltzmann distribution) of Eu(+tfc)$_3$(H_2O)(tmpo) should coexist when two equivalent tmpo ligand is introduced. This would explain the small difference between the experimental spectra of the two and three equivalent cases. The small amount of Eu(+tfc)$_3$(H_2O)(tmpo) also explains the short lifetime component seen in the experiment in toluene. Besides, Supplementary Figure 19 shows that there are two stable configurations in Eu(+tfc)$_3$(tmpo)$_2$. In the most stable configuration, the complex is stabilized by a π stacking interaction between aromatic rings in adjacent tmpo ligands. On the other hand, in the second most stable configuration, the two tmpo ligands avoid to each other to reduce their repulsion. The existence of two stable configurations would be a cause of formation of the amorphous structure and multiple lifetime components in the present material.

Supplementary References

1. Chen, Q.; Zeng, J.; Yan, X.; Huang, Y.; Du, Z.; Zhang, K.; Wen, C. Mild and efficient oxidation of phosphorus(III) compounds with Selectfluor. *Tetrahedron Lett.* **57**, 3379 (2016).

2. Yamagiwa, N.; Tian, J.; Matsunaga, S.; Shibasaki, M. Catalytic Asymmetric Cyano-Ethoxycarbonylation Reaction of Aldehydes using a YLi_3Tris(binaphthoxide) (YLB) Complex: Mechanism and Roles of Achiral Additives. *J. Am. Chem. Soc.* **127**, 3413 (2005).

3. Alyea, E. C.; Malito, J. Non-Metal Derivatives of the Bulkiest Known Tertiary Phosphine, Trimesitylphosphine. *Phosphorus, Sulfur, Silicon Relat. Elem.* **46**, 175 (1989).

4. Hasegawa, Y.; Miura, Y.; Kitagawa, Y.; Wada, S.; Nakanishi, T.; Fushimi, K.; Seki, T.; Ito, H.; Iwasa, T.; Taketsuga, T.; Gon, M.; Tanaka, K.; Chujo, Y.; Hattori, S.; Karasewa, M.; Ishii, K. Spiral Eu(iii) Coordination Polymers with Circularly Polarized Luminescence. *Chem. Commun.* **54**, 10695 (2018).

5. Binnemans, K. Interpretation of europium(III) spectra. *Coord. Chem. Rev.* **295**, 1 (2015).

6. S. Wada et al. Electronic chirality inversion of lanthanide complex induced by achiral molecules. *Sci. Rep.* **8**, 16395 (2018).

7. Lunkley, J. L.; Shirotani, D.; Yamanari, K.; Kaizaki, S. & Muller, G. Chiroptical spectra of a series of tetrakis((+)-3-heptafluorobutylrylcamphorato)lanthanide(III) with an encapsulated alkali metal ion: Circularly polarized luminescence and absolute chiral structures for the Eu(III) and Sm(III) complexes. *Inorg. Chem.* **50**, 12724 (2011).

8. C. Bannwarth, S. Ehlert, S. Grimme, *J. Chem. Theory Comput.* **15**, 1652 (2019).

9. F. Neese, *WIREs Comput. Mol. Sci.* **8**, e1327 (2018).

10. S. Maeda, Y. Harabuchi, M. Takagi, K. Saita, K. Suzuki, T. Ichino, Y. Sumiya, K. Sugiyama, Y. Ono, *J. Comput. Chem.* **39**, 233 (2018).

11. S. Maeda, K. Ohno, K. Morokuma, *Phys. Chem. Chem. Phys.* **15**, 3683 (2013).

12. Gaussian 16, Revision C.01, M. J. Frisch, G. W. Trucks, H. B. Schlegel, G. E. Scuseria, M. A.

Robb, J. R. Cheeseman, G. Scalmani, V. Barone, G. A. Petersson, H. Nakatsuji, X. Li, M. Caricato, A. V. Marenich, J. Bloino, B. G. Janesko, R. Gomperts, B. Mennucci, H. P. Hratchian, J. V. Ortiz, A. F. Izmaylov, J. L. Sonnenberg, D. Williams-Young, F. Ding, F. Lipparini, F. Egidi, J. Goings, B. Peng, A. Petrone, T. Henderson, D. Ranasinghe, V. G. Zakrzewski, J. Gao, N. Rega, G. Zheng, W. Liang, M. Hada, M. Ehara, K. Toyota, R. Fukuda, J. Hasegawa, M. Ishida, T. Nakajima, Y. Honda, O. Kitao, H. Nakai, T. Vreven, K. Throssell, J. A. Montgomery, Jr., J. E. Peralta, F. Ogliaro, M. J. Bearpark, J. J. Heyd, E. N. Brothers, K. N. Kudin, V. N. Staroverov, T. A. Keith, R. Kobayashi, J. Normand, K. Raghavachari, A. P. Rendell, J. C. Burant, S. S. Iyengar, J. Tomasi, M. Cossi, J. M. Millam, M. Klene, C. Adamo, R. Cammi, J. W. Ochterski, R. L. Martin, K. Morokuma, O. Farkas, J. B. Foresman, and D. J. Fox, Gaussian, Inc., Wallingford CT, 2016.
