# Supplementary material for: Chiral lanthanide lumino-glass for a circularly polarized light security device
Source: Commun Chem. 2020 Aug 25;3:119. doi: 10.1038/s42004-020-00366-1 (PMC9814105; doi:10.1038/s42004-020-00366-1)
Supplement: Supplementary file 2 — Description of Additional Supplementary Files [file 42004_2020_366_MOESM2_ESM.pdf]

Description of Additional Supplementary Files

File Name: Supplementary Data 1
Description: Calculation data of the most stable Eu(+tfc)$_3$(H_2O)(tmpo) structure

File Name: Supplementary Data 2
Description: Calculation data of the most stable Eu(+tfc)$_3$(tmpo)$_2$ structure

File Name: Supplementary Data 3
Description: Calculation data of the second most stable Eu(+tfc)$_3$(tmpo)$_2$ structure
