# Supplementary material for: Chiral lanthanide lumino-glass for a circularly polarized light security device
Source: Commun Chem. 2020 Aug 25;3:119. doi: 10.1038/s42004-020-00366-1 (PMC9814105; doi:10.1038/s42004-020-00366-1)
Supplement: Supplementary file 3 — Supplementary Data 1 [file 42004_2020_366_MOESM3_ESM.pdf]

(a) The most stable Eu(+tfc)$_3$(H_2O)(tmpo) structure

Eu	8.769221893622	8.367320858551	10.275398772483
P	6.428828256097	6.417564460142	8.167488321163
F	7.067433923409	11.726056837608	7.120878663519
F	8.810641828278	11.900046241678	5.806947991231
F	8.745491553468	13.005713797026	7.695695341068
F	13.055931515576	8.410893357501	14.034976561968
F	11.200562359060	9.074382934437	14.993949976708
F	12.084465345876	10.253209557446	13.374017213324
F	5.372874379081	9.056292403155	14.441603920464
F	7.190228545415	8.766101043618	15.636545778482
F	6.437926901624	7.152501198785	14.369549232053
O	8.286018934109	9.737409985784	8.285191529386
O	10.749233585318	8.397044193172	8.916598205710
O	10.669764670472	8.805403627262	11.719865318686
O	9.490017322233	6.220207385067	11.063735366491
O	7.785842387358	8.140445818820	12.379206603242
O	8.863711606518	10.655432004551	11.057905202004
O	7.021803380086	7.064166193195	9.440729228133
C	8.420124206500	11.822984677272	7.109330052914
C	9.082323536789	10.632248766550	7.837507473672
C	10.473143342557	10.615515336979	7.946647496363
C	11.196618901593	9.496942384973	8.485443427752
C	12.670982258171	9.903498163281	8.532481931862
C	12.651951988597	10.946549506624	9.719366653862
C	11.916080275820	12.187761934571	9.122757473069
C	11.519627625344	11.693568732884	7.693321282915
C	12.767439654872	10.839526185218	7.269363661882
C	13.644148658593	8.749891929567	8.699067915295
C	12.560145287579	10.091298329424	5.940188133263
C	14.080232778434	11.636831701747	7.170899179581
C	11.848162866307	8.992658941333	13.795338128507
C	11.011192032746	8.183730865720	12.772507371459
C	10.717151857691	6.848963634981	13.080435692520
C	10.012029934145	5.975479657752	12.190483607011
C	9.923840689669	4.613922064981	12.893598020869

C	8.882663821332	4.909850428936	14.041271939324
C	9.659164667807	5.820364637387	15.043054114910
C	11.027723461652	6.018544554220	14.320392386511
C	11.288283623520	4.605570347847	13.684836106777
C	9.584916970370	3.445583489058	11.983068298873
C	12.529032760361	4.571536404062	12.770972930715
C	11.420137460699	3.460251471881	14.705457178478
C	6.621144754455	8.490847913405	14.433360637968
C	7.461010567592	9.024285236278	13.246860171451
C	7.730268058173	10.389992806855	13.215351483366
C	8.393119909768	11.093107973036	12.133645787704
C	8.426308971836	12.575150115672	12.535568934901
C	9.472354664174	12.603537585038	13.709200745924
C	8.745387679982	11.882687086686	14.888646409948
C	7.388064210178	11.465983198912	14.241070019965
C	7.053897312403	12.680904409975	13.305760422476
C	8.704054653350	13.534226824726	11.390935955746
C	5.830774839406	12.432215004750	12.398420606298
C	6.841610268250	14.011070920502	14.050118463156
C	7.379182785574	7.130326577576	6.773744556075
C	6.909035686614	8.156500114569	5.915761118675
C	7.801233482974	8.826514581800	5.048744526951
C	9.159601023221	8.481526038034	5.053580612624
C	9.662838225379	7.492954967873	5.912594989151
C	8.773615270946	6.841224196918	6.787425944170
C	6.590612281407	4.597104909512	8.264289043153
C	6.189893194616	4.059682806555	9.511975947265
C	6.474365748550	2.719964482944	9.848052666634
C	7.124604540674	1.910271576421	8.907270442676
C	7.472772723878	2.402350017754	7.635365932336
C	7.190201293285	3.744083962939	7.300433777440
C	4.631608677870	6.724108227329	8.026461502263
C	3.829948143224	7.352693554956	9.013072919561
C	2.449060666475	7.547253327586	8.771729716827
C	1.883683869397	7.115886659347	7.562005213115
C	2.651470637424	6.470186844952	6.578289678810

C	4.018985893820	6.262084284025	6.835952706016
H	12.136804211818	10.519053027803	10.606914683826
H	13.698080173384	11.177518924246	10.020727036682
H	11.008811463549	12.454379293746	9.702105668144
H	12.570377012835	13.086160785854	9.078287350881
H	11.228547807360	12.495095339248	6.985609327837
H	13.400693532019	8.180336787825	9.623663494524
H	14.692899254184	9.115293052193	8.775593829469
H	13.581671805453	8.039803500825	7.843209415011
H	13.401812625491	9.388414508271	5.742826521031
H	12.515220883839	10.810321438326	5.090188953125
H	11.614144984748	9.509627578300	5.938598043494
H	14.362681976511	12.158934002269	8.108916463163
H	14.000489049372	12.407369019012	6.369523639809
H	14.924642527885	10.961652017376	6.902128995808
H	7.983576736561	5.416097882577	13.637461550390
H	8.550285394418	3.952606991730	14.502056824894
H	9.156077672003	6.794978391210	15.210272807638
H	9.787531974795	5.336458190761	16.036539828988
H	11.841306020911	6.403720289764	14.965679331124
H	8.634932011970	3.634186210769	11.436035533916
H	9.475563596542	2.501311236066	12.563366017931
H	10.375327154084	3.293320012772	11.213690942665
H	12.615319749945	3.586289650584	12.257694271450
H	13.455539637418	4.719226964237	13.371800022561
H	12.506299641363	5.364000486129	11.991043930401
H	10.543826019705	3.350517236161	15.378263496854
H	12.316355878333	3.622572621433	15.347873919761
H	11.557315373459	2.486740005328	14.181175632086
H	10.412730530728	12.094860780667	13.409344193947
H	9.731333818051	13.658505206201	13.951921209491
H	9.299500762566	10.990867112378	15.247720514232
H	8.592089743121	12.557060050753	15.760163818879
H	6.593755831975	11.202519095182	14.966128981428
H	9.725611515714	13.367115669696	10.984584210635
H	8.637699427483	14.592042539715	11.732058690088

H	7.991847930219	13.383455138507	10.550011971614
H	5.680811845272	13.291857369494	11.705149404369
H	4.908232852538	12.335072116743	13.016612344156
H	5.929080919745	11.511652782909	11.779846753084
H	7.694250935158	14.311136454634	14.695334963169
H	5.939097304983	13.940960184387	14.700608327175
H	6.666250977348	14.839610675415	13.326444724098
O	5.585484271708	8.462053545171	5.995723289870
H	7.450276941482	9.636920841129	4.395419354756
H	9.851087041744	9.023288702510	4.386778261109
H	10.738379607683	7.269240065097	5.939761593795
O	9.157872488506	5.939960586945	7.728004932258
O	5.487375333703	4.885804397609	10.343679103886
H	6.162326690529	2.324930045714	10.827186961300
H	7.348517191390	0.858389486762	9.155015558470
H	7.949753994911	1.731109994078	6.905591588911
O	7.474090904054	4.288117785525	6.086116173393
O	4.441690973538	7.759697423720	10.161788013480
H	1.814194687511	8.040139701597	9.521567112269
H	0.806834205447	7.280815626014	7.385285164823
H	2.187715516913	6.122805025878	5.643185022963
O	4.877916514064	5.620186773264	6.000909410774
C	5.071368270758	9.578871443535	5.283949435992
H	5.595310202960	10.518999016529	5.572558893734
H	5.143702003158	9.432847088624	4.178357690485
H	4.001156603761	9.644274116778	5.571598192912
C	10.514703041907	5.515554047780	7.835355625789
H	10.519795981812	4.786264913566	8.670382724234
H	10.854345063401	5.023988646984	6.891558273502
H	11.174191548496	6.373050973127	8.095908407254
C	6.073670390257	5.192147079122	11.618670259315
H	7.094918127192	5.607051763776	11.481026596914
H	5.435558243285	5.974305705709	12.073338570610
H	6.100706595017	4.296574138282	12.285027837489
C	8.398903354916	3.637679296334	5.230988257854
H	9.337911897397	3.368275931996	5.771743987553

H	7.965786538250	2.715899118792	4.770299229850
H	8.637075290413	4.369521015073	4.430405457432
C	3.683009772637	8.353928215069	11.214514998182
H	2.897681517477	7.653097180412	11.582091944634
H	4.407450988226	8.560589181987	12.028542497636
H	3.217683719369	9.314664301012	10.891002225064
C	4.546289041854	5.434644113713	4.636772969352
H	3.715064398000	4.699668856181	4.507896400424
H	4.266225371692	6.403928054973	4.158598763478
H	5.465400156002	5.034983673577	4.161556040964
O	6.469113981303	9.646380631518	10.203283577019
H	5.899602692330	8.848861757918	10.033761823437
H	6.749930898096	9.975036642428	9.307442541333
