# Supplementary material for: Chiral lanthanide lumino-glass for a circularly polarized light security device
Source: Commun Chem. 2020 Aug 25;3:119. doi: 10.1038/s42004-020-00366-1 (PMC9814105; doi:10.1038/s42004-020-00366-1)
Supplement: Supplementary file 4 — Supplementary Data 2 [file 42004_2020_366_MOESM4_ESM.pdf]

(b) The most stable Eu(+tfc)$_3$(tmpo)$_2$ structure

Eu	10.000718792755	9.029248881402	10.618046548069
P	8.766665774091	6.003392415260	8.539825086749
P	13.667359003949	8.232462153912	9.596208469744
F	12.742516927558	12.638128556406	12.672703963282
F	11.719178389753	14.218161696514	11.562775735509
F	13.632661713104	13.448801183127	10.844267924570
F	5.683693808778	8.281795068994	14.034660267312
F	5.179103226679	6.697896543534	12.615749803730
F	5.244695066233	8.785039295103	11.956347833534
F	6.987415584574	13.772142449044	10.896370268156
F	5.500580936170	12.250385953654	11.415747759561
F	6.428241716832	12.243718514518	9.438827839926
O	11.413988150951	10.931405711058	11.193495890959
O	10.307036238539	10.288311646034	8.535900657587
O	7.792798723669	8.448074087342	11.451456426175
O	10.308171025935	7.146755075401	12.181823434953
O	8.461645640315	10.890539305350	10.414841028731
O	9.729280578034	9.822169525256	12.924596272473
O	9.269713642924	7.306777623261	9.167282846013
O	12.280266997114	8.355804740392	10.230013860164
C	12.450409231939	13.076106726191	11.428553059576
C	11.696939309976	12.008286862665	10.590337729227
C	11.376742741172	12.350801205939	9.271851292285
C	10.710717308908	11.467811124081	8.356967754305
C	10.621165225838	12.235085457023	7.015113207698
C	12.113259697554	12.246603580672	6.519798420227
C	12.829993361570	13.233224439565	7.494670473836
C	11.688172860895	13.618066961220	8.483292466184
C	10.432595468851	13.706849027458	7.552263889702
C	9.604821567207	11.725784706999	6.008192073094
C	9.118072119967	13.902571703092	8.331613562371
C	10.518894171437	14.793696857835	6.465872909884
C	5.861790621060	7.879949569592	12.741251112558
C	7.364386193226	7.719965381945	12.392038593266
C	8.105688436134	6.822178255901	13.169728790377

C	9.514278661545	6.613744304629	13.000471358828
C	9.914161109162	5.526041275613	14.028663567524
C	9.270344709138	4.230498651076	13.410593081019
C	7.734858154816	4.436292398572	13.619352832059
C	7.665603249677	5.855416505768	14.263195381026
C	8.916784312328	5.865247614709	15.202618725811
C	11.388298365789	5.434772444997	14.386614099079
C	9.190273150223	7.241720760153	15.833697655026
C	8.875311663793	4.817324505781	16.329591140539
C	6.672548382646	12.451554516040	10.754798783175
C	7.804656129403	11.538102705543	11.293890623665
C	8.022901542655	11.555343644282	12.672593558130
C	9.011282283734	10.757609122380	13.354076759109
C	8.997687537883	11.212942418696	14.825104410006
C	7.645694934551	10.608028780752	15.355812129322
C	6.544341306159	11.465996415623	14.654721969103
C	7.383681206762	12.422958809757	13.754746917120
C	8.630038818385	12.737349181612	14.653861427241
C	10.255311365602	10.861566582054	15.603429775634
C	9.708507336249	13.550185201282	13.917062743092
C	8.300472424250	13.461922914652	15.971865509931
C	10.138390641155	5.016521526201	7.813443950692
C	10.155288798090	4.461795117015	6.507098478942
C	11.076018482098	3.448621572102	6.160635301753
C	12.058426222903	3.072536946887	7.087964949768
C	12.165930021389	3.704611970984	8.332697510954
C	11.219877795451	4.692155600164	8.687473149188
C	7.790868764554	4.909676102731	9.695899981224
C	6.468091298868	5.359723885579	9.981049518031
C	5.478654260419	4.483193085793	10.479770776395
C	5.824935131768	3.158667169638	10.768484452405
C	7.138202925241	2.701113606957	10.589026130423
C	8.119989699555	3.575290570702	10.070521819818
C	7.470442443005	6.285014168547	7.245531861304
C	7.017252578466	7.590997671931	6.913597159832
C	5.833893059174	7.760339303161	6.150485777202

C	5.117518294941	6.641074659187	5.711864540045
C	5.551843332664	5.337895150277	6.012252861574
C	6.730479116888	5.171799683503	6.766149393337
C	13.733372576477	6.932317697096	8.293004738155
C	14.719373260324	5.913627356025	8.197939474188
C	14.896612825297	5.180325359005	7.004723026750
C	14.006996969872	5.382832410841	5.940253780058
C	12.931436611875	6.271127084087	6.051179924686
C	12.780102923992	7.030851816672	7.233350053923
C	14.359588395635	9.814764096311	8.881483853283
C	14.706333344086	10.030250792552	7.516133919125
C	15.613960895216	11.047593411907	7.144718145429
C	16.159821850985	11.885053270093	8.127104504715
C	15.786431143903	11.754902629041	9.469003659455
C	14.864691444884	10.748920115880	9.833017837224
C	14.988585348039	7.881520432240	10.853628918666
C	14.688316659871	7.686722404124	12.223735890758
C	15.728653190908	7.635386212812	13.183485059472
C	17.061529189106	7.785249531649	12.790788546642
C	17.388276649969	7.972514387199	11.433724360892
C	16.357681485784	8.000701088390	10.475579079485
H	12.537953349852	11.220029762484	6.546931971660
H	12.157448457916	12.593829073678	5.462449609037
H	13.669136136434	12.762960310013	8.041444353272
H	13.235045402616	14.122684144683	6.961764934657
H	11.888833369233	14.522690445242	9.089760904142
H	9.715844241678	10.634633675885	5.837049496911
H	9.720874097893	12.247911000404	5.031135949947
H	8.570064792244	11.901287947793	6.369972844498
H	8.239288614196	13.903900387491	7.647614733989
H	9.124260021202	14.877252247168	8.870186603064
H	8.952024547402	13.103810462841	9.083634780740
H	11.416339131039	14.716554772656	5.815521306154
H	10.535150280091	15.802477744247	6.939771697762
H	9.624232527925	14.752534542003	5.803107032210
H	9.551980068301	4.127086589207	12.339816754523

H	9.650919751957	3.328142735548	13.941383460444
H	7.167801511082	4.419458575092	12.669863739534
H	7.297471862184	3.656874465930	14.282987644438
H	6.702063839398	6.090494089705	14.756500286944
H	11.567841409282	4.590552160287	15.091243904479
H	11.742228206378	6.371002058803	14.870532106536
H	12.022081000437	5.284223447970	13.491172976673
H	10.122375423917	7.225079887605	16.443965615466
H	8.354262651915	7.540485930802	16.506388762565
H	9.301062152134	8.033423395917	15.063003521495
H	8.737349561561	3.773992795197	15.974412996094
H	8.036926912153	5.045788098067	17.027934506397
H	9.818092903548	4.843156184476	16.922640050340
H	7.564679671258	9.531762385262	15.100903198011
H	7.606196752937	10.684448018865	16.465843694921
H	5.855047944289	10.852277104669	14.040492404891
H	5.927428294126	12.032335247851	15.387586667212
H	6.835311457093	13.313036153040	13.389662365498
H	10.421386702174	9.763196024993	15.614608042386
H	10.185518494202	11.214181139637	16.657705672555
H	11.150555257784	11.323590914145	15.130222624202
H	10.630114723221	13.652985531476	14.533006855630
H	9.336946707884	14.575655297064	13.690203042534
H	9.999956909111	13.084208862905	12.954490068574
H	7.573751128057	12.924999977302	16.617551424022
H	7.874881499680	14.469092323522	15.754913218161
H	9.226675634027	13.614669136340	16.572166185322
O	9.303601894081	5.005351716103	5.592504466287
H	11.068650286733	3.005911079454	5.153743819558
H	12.797968223939	2.302139032368	6.808747435597
H	12.987442116135	3.450023689061	9.016096187990
O	11.235411141303	5.358380252130	9.851237312338
O	6.213370113016	6.662999153497	9.710064257462
H	4.463551378474	4.849656897306	10.683146877505
H	5.063539219722	2.473695301638	11.179178740143
H	7.398907161813	1.672940939647	10.876236120772

O	9.406020849828	3.180662465270	9.892374378201
O	7.732151510653	8.639726438953	7.372099084776
H	5.482049617097	8.773173873206	5.905213198367
H	4.197301531479	6.782928626816	5.118987678731
H	4.985494408752	4.466301549124	5.651930658711
O	7.236455062397	3.941518831733	7.083829551428
O	15.425741954524	5.627105660254	9.327775043882
H	15.676551439904	4.408246468744	6.932664102003
H	14.121813443483	4.786980722451	5.018382167517
H	12.198443788051	6.356902754888	5.237808999234
O	11.780302977451	7.903819711020	7.429418900286
O	14.138042434703	9.212572844569	6.594173092011
H	15.874389351120	11.207478805914	6.089175165180
H	16.861788751331	12.683297163165	7.831227352891
H	16.165933116202	12.458641505225	10.221859870878
O	14.443625110864	10.590199458368	11.114233613843
O	13.404117999480	7.521619905334	12.651563615766
H	15.452176191827	7.470538744040	14.237204185049
H	17.867801567261	7.748220612996	13.543638593299
H	18.440651598488	8.073129589840	11.128324173488
O	16.597040452624	8.124924210912	9.135930007489
C	8.911614947492	4.254078717537	4.456598219466
H	9.720621460537	4.207644189559	3.686803997139
H	8.609881028476	3.219199228694	4.743710547364
H	8.033168167854	4.785386301652	4.031935345563
C	12.240040335024	5.096434817206	10.825623146808
H	12.122785407136	5.904569638730	11.571726744395
H	12.076853583688	4.093334034011	11.290080363745
H	13.262449515535	5.147953593402	10.388969963860
C	4.875852893952	7.072366805197	9.436985956965
H	4.241166793251	7.033070226482	10.349196404015
H	4.952107598501	8.126024058664	9.105067831574
H	4.437087241511	6.451384520368	8.620434590695
C	9.792092523052	1.843244019001	10.149840865242
H	9.690676530379	1.589858211580	11.233025270604
H	9.202930480152	1.116610501204	9.538851682721

H	10.859889132644	1.784950746389	9.851169759584
C	7.084150380062	9.887197066759	7.619593358860
H	6.136062998460	9.737242566770	8.184446827095
H	7.781471939005	10.467339510486	8.254147342971
H	6.871034864705	10.435779877748	6.671660821437
C	6.365752046110	2.820788022991	7.128254004625
H	5.454763630479	3.045623689386	7.731862050169
H	6.071202074830	2.480390580216	6.105136289672
H	6.938109895576	2.014195259995	7.631708453316
C	16.683954181156	4.982579846459	9.233285833222
H	16.579020486112	3.893092146560	9.007455002240
H	17.326040280257	5.463160603330	8.457528586019
H	17.160691662359	5.105176149711	10.229354871325
C	10.821640379289	8.141852468093	6.402445828828
H	10.064980022348	8.797058990147	6.870970664314
H	11.304013295994	8.653509694910	5.534043375831
H	10.336640188267	7.198330535258	6.064936511865
C	14.550225714356	9.259643702402	5.239629110048
H	14.264131411866	10.226306738022	4.758889897178
H	15.651111679082	9.100493580458	5.137382509397
H	14.016826525716	8.422880781484	4.741988675768
C	15.274595596698	11.050360210805	12.178767949096
H	16.311480258536	10.655904057044	12.060995303517
H	15.277244302690	12.160831802285	12.241144105596
H	14.825356746783	10.641758094214	13.104649007078
C	12.796005760643	8.658582915859	13.293172068614
H	12.737878031568	9.520983321623	12.593898401686
H	11.763555374252	8.357545740507	13.546557770795
H	13.352403088980	8.938225582203	14.222467212643
C	17.824577088106	8.680095778242	8.691314355912
H	18.039786700217	9.644056310170	9.210732963526
H	18.678453532378	7.972530633627	8.833088480712
H	17.689099438193	8.881193570034	7.608321585874
