# Supplementary material for: Chiral lanthanide lumino-glass for a circularly polarized light security device
Source: Commun Chem. 2020 Aug 25;3:119. doi: 10.1038/s42004-020-00366-1 (PMC9814105; doi:10.1038/s42004-020-00366-1)
Supplement: Supplementary file 5 — Supplementary Data 3 [file 42004_2020_366_MOESM5_ESM.pdf]

(c) The second most stable Eu(+tfc)$_3$(+tmpo)$_2$ structure

Eu	10.158138227184	8.714719574955	10.478349346553
P	7.606640120874	7.083165455754	8.071260496908
P	13.873730124085	8.616862760384	11.411521234851
F	9.820611191918	13.702132335869	9.813618098523
F	9.346993748534	13.604399082688	7.688841970678
F	11.418688636661	13.871745887092	8.330309002278
F	9.376253479685	6.617530384981	15.699463395075
F	7.725391892937	5.607658150836	14.693598995685
F	7.762510696665	7.789894596952	14.812344723938
F	5.678346378380	11.810241802763	11.097193969943
F	5.404448216951	10.753275044212	12.999419944684
F	5.344264835303	9.646116512908	11.136114254717
O	10.172578881136	11.088889139161	9.758110653188
O	11.469606612590	8.854014315054	8.363927330731
O	9.214401777827	7.865666274911	12.632127124159
O	10.491770773125	6.278207568347	10.551453827885
O	7.967002311679	9.676894772331	10.896863327823
O	10.486184271018	10.230948453235	12.406513735979
O	8.788570574774	7.836261247142	8.705846093520
O	12.361201494289	8.359572108426	11.322622418902
C	10.252429517787	13.216658709019	8.636024021265
C	10.472154112820	11.673666354514	8.673047584536
C	10.986323464335	11.086067397770	7.513145370511
C	11.440889601443	9.722917358907	7.453582844163
C	11.953446963897	9.521183631589	6.003936741352
C	13.256469183254	10.392050426679	5.927830527143
C	12.742810900834	11.863317146508	5.949015354624
C	11.206692299475	11.673368649130	6.124132948445
C	10.910217291331	10.417571623630	5.231105125917
C	12.122933382882	8.081190583645	5.550412159908
C	9.471482637227	9.879840891907	5.341884780676
C	11.222593128252	10.626102104023	3.737492180493
C	8.540630273138	6.706153786554	14.621699352147
C	9.304542138559	6.771842271555	13.264025416096
C	9.987993853980	5.624165281717	12.848205065331

C	10.493748105337	5.466841501918	11.510692377237
C	10.987204107412	3.999719352759	11.424406286852
C	9.669143141178	3.151642860326	11.463552276045
C	9.160356649774	3.300966637398	12.929311385782
C	10.203902452126	4.285738281568	13.544440205678
C	11.551125741452	3.816864961228	12.882093553949
C	11.899065399623	3.697420323923	10.256109821178
C	12.757501588656	4.732855051616	13.170379891479
C	11.959121130570	2.373552959367	13.230557393344
C	5.978916673686	10.654628105261	11.766842301407
C	7.513722226362	10.428847770269	11.820148651532
C	8.230339708295	11.079790498702	12.828159582202
C	9.644118010164	10.904462856704	13.043340273357
C	9.977897108255	11.677470164005	14.342579545145
C	9.275615863548	10.788980014505	15.440724895624
C	7.750827579524	11.058425896270	15.242045249893
C	7.744431688132	11.967016540157	13.976349886876
C	8.987771169600	12.889918866663	14.210280232515
C	11.453978955980	11.959315263523	14.573533980976
C	9.288881137706	13.794912549659	13.001479673550
C	8.912715305284	13.774635184553	15.467586969678
C	7.883469575435	6.908969719620	6.251935894204
C	7.042235528175	7.441529772471	5.234154816684
C	7.367809438276	7.271850547931	3.866422977089
C	8.522520281073	6.566387272856	3.508319102646
C	9.370132163263	6.024420077586	4.485757327191
C	9.050254280379	6.209308383075	5.846759734056
C	7.345173543291	5.414510522649	8.828922243983
C	6.961879672255	5.457373133131	10.199794057416
C	6.412190225590	4.327335736390	10.845141668609
C	6.313232025209	3.122727474657	10.139775950474
C	6.721890323946	3.029708820273	8.801124770629
C	7.212669504164	4.179394868008	8.139510212097
C	5.990141978984	7.946941710713	8.287019617232
C	5.986540001667	9.365115018643	8.185210336122
C	4.799565632813	10.105941189304	8.374715680659

C	3.603798194188	9.426850704128	8.636327403929
C	3.556175883932	8.026228797532	8.662935592425
C	4.741568539980	7.288115460730	8.444557329870
C	14.815467997671	7.036182208580	11.429863142387
C	15.784389639103	6.629587952762	12.383245448971
C	16.465052660281	5.396729711032	12.226155648502
C	16.215012090802	4.604603916804	11.096621334246
C	15.279102096220	4.989807769806	10.124147102620
C	14.561744673529	6.188360976624	10.319136948105
C	14.480722606968	9.649962839722	10.004074171591
C	15.574037880124	9.368529544924	9.142566009017
C	16.100315231210	10.373063448104	8.296677412595
C	15.530405499404	11.655662459109	8.303609621031
C	14.436936193202	11.960965652485	9.122688976534
C	13.915239074955	10.954147600378	9.961659167030
C	14.329108818114	9.530925931077	12.953260512133
C	13.723375895580	9.076345030617	14.155594028291
C	13.936982625023	9.742537641683	15.382720247020
C	14.784475848193	10.857181795715	15.414101559840
C	15.453217453682	11.290962807184	14.260794449277
C	15.259374802787	10.600981029269	13.041055135518
H	13.930420479470	10.156802641961	6.777975056797
H	13.813766159905	10.152632928436	4.993694477961
H	13.160473272469	12.448743481844	6.791801440887
H	12.985782840081	12.406528793596	5.008496303339
H	10.602334173344	12.571071637921	5.887653285875
H	11.296694953714	7.451148898714	5.936475574706
H	13.082708199506	7.660346677262	5.918017667875
H	12.131736440732	8.014892572898	4.438977772547
H	9.359999040779	8.967204511197	4.716491719449
H	8.737278049953	10.632214692244	4.970478633333
H	9.177937755184	9.599892563158	6.375736945549
H	12.271732102729	10.922913946684	3.526209555602
H	10.560174379565	11.421972736007	3.324283366647
H	11.017274182424	9.695393044536	3.160765710894
H	8.943044869397	3.519207618987	10.709267345506

H	9.896356498887	2.092891036046	11.204908732545
H	8.141580525324	3.733206674315	12.985406866572
H	9.140544200643	2.326884496597	13.467807805183
H	10.206307353132	4.321546140190	14.653205284716
H	12.110941957808	2.607275506864	10.174613816804
H	12.861495715289	4.235991835877	10.361666468237
H	11.417545673100	4.041871798667	9.315803386405
H	13.664650050950	4.368311246656	12.636231826260
H	12.996055198180	4.737545909556	14.258917299428
H	12.598848269116	5.784581719865	12.850889147344
H	11.177134674221	1.613889093201	13.018962089410
H	12.209874900504	2.301433240175	14.314510290981
H	12.869569458490	2.081550250482	12.658570511377
H	9.533714052467	9.716440515791	15.303986329436
H	9.630059077927	11.089192675860	16.452391259906
H	7.171760482644	10.131790083582	15.062810554308
H	7.302218856400	11.572998684410	16.121365838014
H	6.795056373354	12.508380694408	13.798735936617
H	12.070545253003	11.127714523330	14.171668114199
H	11.689609027618	12.077789454599	15.654566990871
H	11.780789801460	12.889054509299	14.057873094901
H	10.246864381893	14.347365405009	13.141353609596
H	8.483743365215	14.555243288959	12.876637331684
H	9.358084524499	13.221657831385	12.053802051205
H	8.756375146720	13.209100867272	16.410574826981
H	8.078504474904	14.508362132072	15.374242312675
H	9.857422105846	14.354217056462	15.584698691738
O	5.922788243037	8.096342514755	5.623227469522
H	6.716027830780	7.688130360518	3.084930835230
H	8.773394801974	6.441338560760	2.440780672427
H	10.284139342472	5.490042787661	4.190118052306
O	9.826075936005	5.726691597775	6.854633747964
O	7.104448742291	6.659600948889	10.803645308402
H	6.083756255109	4.383396568049	11.891410360831
H	5.914824011633	2.227875648626	10.648118599533
H	6.641770256221	2.068743790861	8.272974742679

O	7.528367989259	4.165933178508	6.815475263104
O	7.155932647267	9.930979965058	7.814790246963
H	4.816227956817	11.203941492911	8.367537851025
H	2.680222716083	10.004417912827	8.814313940274
H	2.596652797807	7.512924431212	8.821323329781
O	4.734574538724	5.929680901786	8.319148000137
O	16.037308863971	7.473524583924	13.415301560216
H	17.206511471654	5.067842151492	12.968351754450
H	16.766531779632	3.656459842729	10.971521618700
H	15.100762634493	4.360873171373	9.240079138541
O	13.597195243039	6.644889890615	9.494838887865
O	16.098369004318	8.114094483151	9.190462127459
H	16.942142299871	10.156717471284	7.623740987967
H	15.937386980490	12.430852943011	7.631902765522
H	13.988329086440	12.963359655137	9.106299611114
O	12.897922671997	11.154478267253	10.829564444800
O	12.983270424534	7.950078861489	14.048152302353
H	13.431787060395	9.407494063616	16.299268773945
H	14.937840958553	11.396619895635	16.364682677814
H	16.144729009218	12.143942082047	14.318273428737
O	15.968712542099	10.901864620758	11.919018752079
C	5.215268758930	8.929150612404	4.722955775222
H	5.895829237311	9.660824330315	4.223845971661
H	4.669766558890	8.339674908696	3.945500792581
H	4.479739587834	9.476622592112	5.350228448659
C	10.698903532810	4.646329666739	6.573271012270
H	11.030277974343	4.264775804179	7.558764867732
H	10.168539849601	3.836180698367	6.018545318963
H	11.594002609743	4.975025828893	5.991650503975
C	6.346614569461	6.953092106462	11.974192700501
H	6.515955718145	6.197983492300	12.771009910867
H	6.720979724910	7.928725631319	12.326366744989
H	5.262269047761	7.015188399174	11.722028691458
C	7.230606872107	3.039354205150	6.014155862734
H	7.833952764810	2.144662619302	6.306569297118
H	6.145156263555	2.776783057407	6.054257165706

H	7.499672660832	3.335430614256	4.977760534652
C	7.287577444455	11.343209397596	7.827172067543
H	7.186980816192	11.744515748374	8.860928144958
H	8.303537382501	11.542667673321	7.445513583229
H	6.547872275972	11.834971014167	7.148890067867
C	3.613178393401	5.182553707249	8.755512602402
H	3.351896211684	5.417982084972	9.815483559239
H	2.717754595473	5.352441564907	8.107534986054
H	3.922073359540	4.118484745338	8.688809365646
C	16.843352042018	7.063304781628	14.504028893915
H	16.455820022933	6.126560902866	14.973473851673
H	17.908786338919	6.910145120713	14.202165686760
H	16.787440980311	7.892686829343	15.240361496072
C	13.424428160087	6.129956989180	8.190655544067
H	12.513955621334	6.635757315741	7.815830890721
H	14.306873890930	6.382978707159	7.553384077190
H	13.271007841775	5.026969729217	8.189205252710
C	17.243748331973	7.778977906494	8.431429144698
H	17.054845310557	7.860974495682	7.332669402868
H	18.121979024333	8.415749393683	8.699995094474
H	17.464345848673	6.720481169242	8.685937233741
C	12.542862566685	12.468260027188	11.239407313610
H	13.351852303843	12.900271928348	11.877137727545
H	12.341520212406	13.139230313493	10.379136831191
H	11.613103358220	12.344887650861	11.820767459244
C	12.106590668751	7.588476508312	15.103395287217
H	11.400198601292	8.417754119209	15.336043965741
H	11.534258820616	6.721235315431	14.730057667816
H	12.665116669331	7.292845927581	16.025123401713
C	16.719868761614	12.100769045334	11.846535564514
H	16.088554773552	12.995646691462	12.066399280473
H	17.595572627921	12.087725856838	12.541548715595
H	17.078463837485	12.160753277775	10.798158833870
